# An essential role for miR-15/16 in Treg suppression and restriction of proliferation

**DOI:** 10.1016/j.celrep.2023.113298

**Published:** 2023-10-19

**Authors:** Kristina Johansson, John D. Gagnon, Simon K. Zhou, Marlys S. Fassett, Andrew W. Schroeder, Robin Kageyama, Rodriel A. Bautista, Hewlett Pham, Prescott G. Woodruff, K. Mark Ansel

**Affiliations:** 1 Department of Microbiology and Immunology, University of California, San Francisco, San Francisco, CA 94143, USA; 2 Sandler Asthma Basic Research Center, University of California, San Francisco, San Francisco, CA 94143, USA; 3 Department of Medicine, Division of Pulmonary and Critical Care Medicine, University of California, San Francisco, San Francisco, CA 94143, USA; 4 Department of Medical Biochemistry and Cell Biology, University of Gothenburg, 40530 Gothenburg, Sweden; 5 Department of Internal Medicine and Clinical Nutrition, University of Gothenburg, 40530 Gothenburg, Sweden; 6 Department of Dermatology, University of California, San Francisco, San Francisco, CA 94143, USA; 7 Department of Medicine, Genomics CoLab, University of California, San Francisco, San Francisco, CA 94143, USA; 8 Lead contact

## Abstract

The miR-15/16 family targets a large network of genes in T cells to restrict their cell cycle, memory formation, and survival. Upon T cell activation, miR-15/16 are downregulated, allowing rapid expansion of differentiated effector T cells to mediate a sustained response. Here, we used conditional deletion of miR-15/16 in regulatory T cells (Tregs) to identify immune functions of the miR-15/16 family in T cells. miR-15/16 are indispensable to maintain peripheral tolerance by securing efficient suppression by a limited number of Tregs. miR-15/16 deficiency alters expression of critical Treg proteins and results in accumulation of functionally impaired FOXP3^lo^CD25^lo^CD127^hi^ Tregs. Excessive proliferation in the absence of miR-15/16 shifts Treg fate and produces an effector Treg phenotype. These Tregs fail to control immune activation, leading to spontaneous multi-organ inflammation and increased allergic inflammation in a mouse model of asthma. Together, our results demonstrate that miR-15/16 expression in Tregs is essential to maintain immune tolerance.

## In brief

Tregs are essential suppressors of autoimmunity and allergy. Johansson et al. demonstrate that the miR-15/16 family of microRNAs control core Treg function by limiting their proliferation and expression of the key functional molecules FOXP3, IL-2Rα, CTLA4, PD-1, and IL-7Rα.

## Graphical Abstract

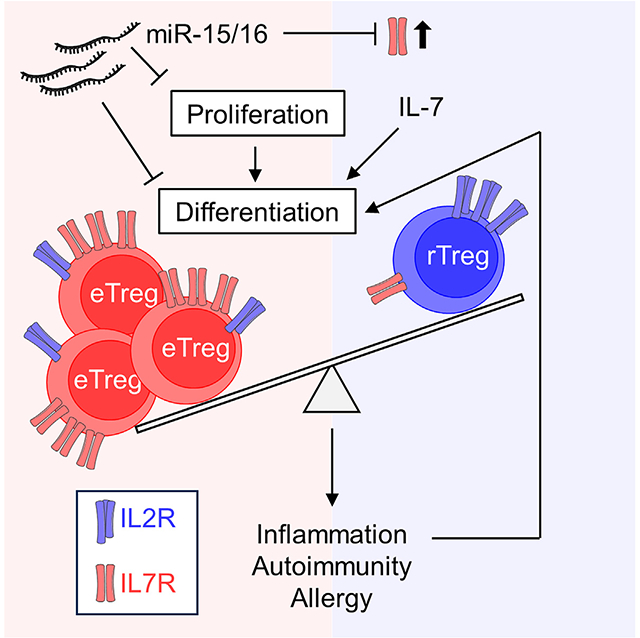

## INTRODUCTION

Regulatory T cells (Treg) are essential mediators of peripheral immune tolerance. Severe functional Treg defects or complete lack of Tregs results in systemic immune activation and lethal autoimmunity.^1–3^ Treg function relies on IL-2 signaling via STAT5, which sustains high expression of FOXP3 and the high-affinity IL-2 receptor α chain, CD25.^1,4–7^ This positive feedback loop reinforces Treg transcriptional identity and promotes maturation of CD4^+^FOXP3^lo^ cells into CD4^+^FOXP3^+^CD25^hi^ Tregs during thymic development.^8–10^ Functionally diverse Treg subgroups depend on expression of specific transcription factors.^11,12^ For instance, gradient expression of TCF1 and LEF1 separates Tregs into subpopulations of resting and activated effector phenotypes with distinct transcriptional profiles.^11^ Functional specialization of Tregs plays a critical role in preventing loss of self-tolerance,^13^ but the mechanisms that govern specification of Treg subgroups are incompletely understood.

MicroRNAs (miRNAs) are short non-coding RNAs that regulate gene expression by binding untranslated regions of target mRNAs to recruit Argonaute (Ago) proteins and promote mRNA degradation and translational repression.^14^ Coordinated regulation of gene networks gives miRNAs a profound biologic impact and it is well known that miRNAs play crucial roles in differentiation and function of T cells.^15–19^

The miR-15/16 miRNA family comprises four abundant miRNAs that are encoded in two separate clusters (miR-15a/miR-16–1 and miR-15b/miR-16–2). miR-15/16 have been studied extensively in lymphocyte malignancies, where they function as tumor suppressors.^20,21^ Deletion of miR-15a/miR-16–1 in the mouse *Dleu2/Mirc30* locus leads to clonal B cell lymphoproliferation that recapitulates many features of chronic lymphocytic leukemia.^20^ In more recent years, a role for miR-15/16 regulation of T effector responses has emerged.^22–26^ miR-15b/16–2 overexpression promotes FOXP3 expression and Treg differentiation,^27^ and a recent report identified a role for miR-15/16 in effector Tregs,^28^ yet our understanding of how miR-15/16 regulate Tregs remains limited.

Here, we generated mice with conditional inactivation of both miR-15/16 clusters in Tregs to determine their impact on Treg development and function. miR-15/16 restricted proliferation of thymus-derived Tregs and regulated the expression of proteins that are fundamental to Treg function, including securing high CD25 expression. Deletion of miR-15/16 in Tregs resulted in lymphoproliferative disease with systemic tissue inflammation and accumulation of Tregs with an activated effector phenotype.

## RESULTS

### Treg-specific miR-15/16 expression is essential to prevent autoimmune inflammation

miR-15/16 are among the most abundant miRNAs occupying Ago2 complexes in resting T cells, but the miRNAs in this family are dynamically expressed. They are downregulated by T cell receptor (TCR) stimulation *in vitro* and remain low during memory T cell formation following lymphocytic choriomeningitis virus infection in mice.^22^ miR-15/16 expression in non-effector T cell subsets such as Tregs is less studied. We analyzed published miRNA expression data from sorted human peripheral blood T cells to assess miR-15/16 miRNAs in distinct effector and non-effector T cell subsets.^29^ Tregs exhibited the highest expression of miR-15a, −15b, and −16 compared with CD4^+^ naive, Th1, Th2, and Th17 cells (Figure S1).

**Figure 1.**
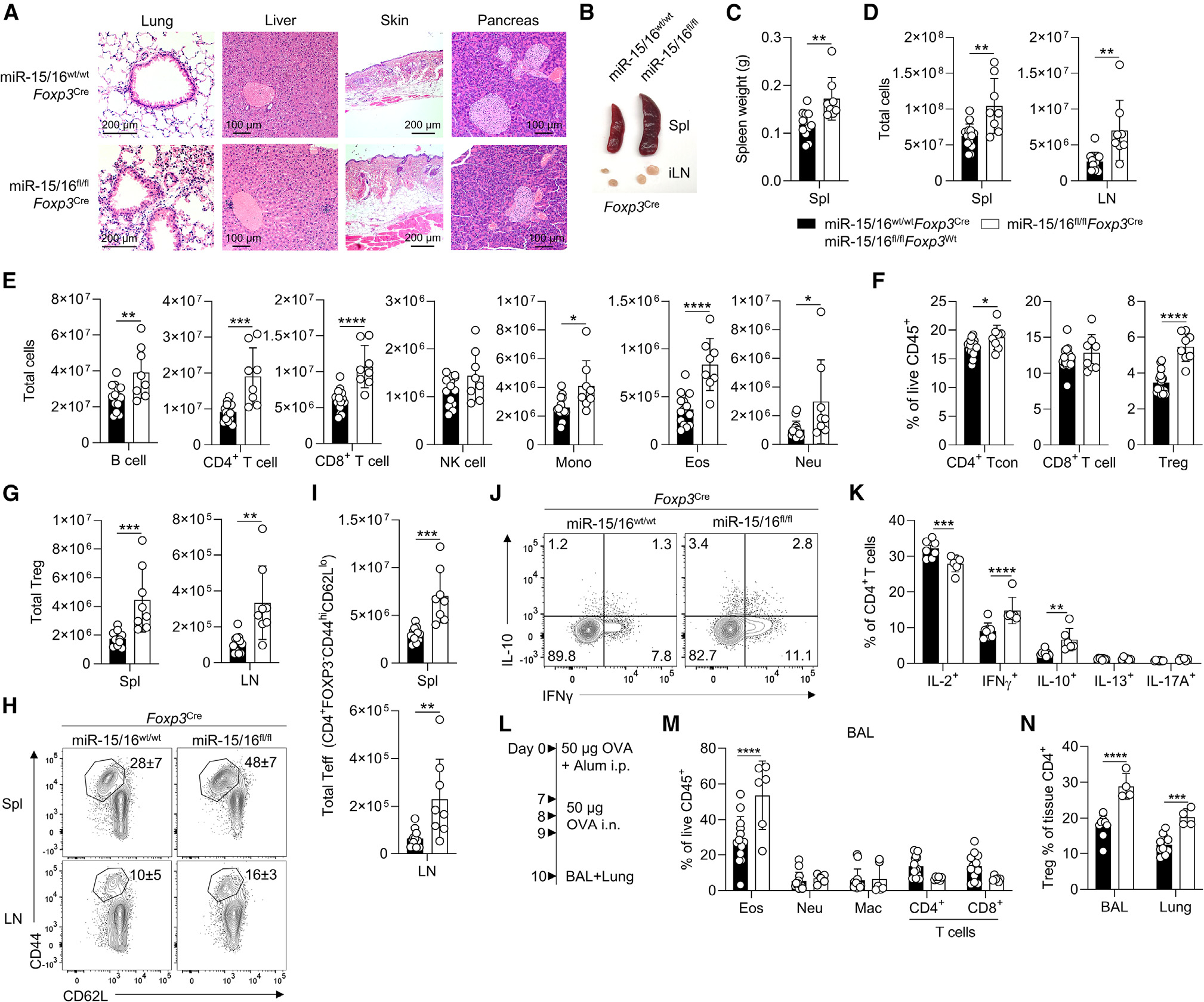
Treg-specific miR-15/16 expression is essential to prevent spontaneous and induced tissue inflammation (A) H&E-stained sections of lung, liver, skin, and pancreas; control mice with miR-15/16-sufficient Tregs (miR-15/16^wt/wt^*Foxp3*^Cre^ or miR-15/16^fl/fl^*Foxp3*^Wt^; “WT”) on the top row and mice with miR-15/16-deficient Tregs (miR-15/16^fl/fl^*Foxp3*^Cre^, “KO”) on the bottom row. Representative image of spleen (Spl) and inguinal lymph nodes (LN) (B), spleen weight quantified (C), and total cells quantified in spleen and lymph nodes (D) from the same mice. Total spleen B cells (CD45^+^CD4^−^CD8^−^NK1.1^−^CD19^+^), CD4^+^ T cells (CD45^+^CD11b^−^CD11c^−^CD8^−^CD4^+^), CD8^+^ T cells (CD45^+^CD11b^−^CD11c^−^CD4^−^CD8^+^), NK cells (CD45^+^CD4^−^CD8^−^CD19^−^NK1.1^+^), monocytes (Mono) (CD45^+^CD11b^int-hi^CD11c^int-lo^NK1.1^−^Ly6G^−^), eosinophils (Eos) (CD45^+^CD11b^+^Siglec-F^+^), and neutrophils (Neu) (CD45^+^CD11b^+^Ly6G^+^) in spleen (E). Percentage of CD4^+^ conventional T cells, CD8^+^ T cells, and Treg in spleen (F), and total Tregs in spleen and lymph nodes (G) of WT and KO mice. Representative contour plots with frequencies (H) and quantification of total (I) CD44^hi^CD62L^lo^ T effector cells in spleen and lymph nodes of WT and KO mice. Intracellular cytokine staining of CD4^+^ T cells from WT and KO mice restimulated *ex vivo*; contour plots show IFN-γ and IL-10 expression (J) and quantification of IL-2^+^, IFN-γ^+^, IL-10^+^,IL-13^+^,and IL-17A^+^ cells (K). (L) Airway inflammation model induced by intraperitoneal (i.p.) OVA sensitization and intranasal (i.n.) OVA challenge. Bronchoalveolar lavage (BAL) and lung tissue were collected 24 h after the last OVA challenge. Frequency of eosinophils (CD11b^+^Siglec-F^+^), neutrophils (CD11b^+^Ly6G^+^), alveolar macrophage (Mac) (CD11c^+^CD11b^lo^), CD4^+^ T cells (CD4, CD11b^−^CD11c^−^CD4^+^), and CD8^+^ T cells (CD8, CD11b^−^CD11c^−^CD8^+^) among live hematopoietic cells in BAL (M), and frequency of tissue-resident Tregs in BAL and lung tissue of OVA-challenged WT and KO mice (N). Twenty-week-old unchallenged mice in (A–K). Data from 7 independent experiments. N = 6–14 mice/group. In (C–G) and (I) unpaired t test two-tailed and two-way ANOVA with Bonferroni’s multiple comparison test in (K, M, and N). Bar graphs are shown with error bars demonstrating standard deviation. *p < 0.05, **p < 0.01, ***p < 0.001, and ****p < 0.0001 for significance.

To investigate the functional importance of miR-15/16 in Tregs specifically, we generated mice with conditional deficiency of miR-15 and miR-16 using Cre recombinase driven by the endogenous *Foxp3* locus (*Foxp3*^Cre^). *LoxP*-flanked miR-15a/16–1 (*Mirc30*) and miR-15b/16–2 (*Mirc10*) alleles (hereafter referred to as miR-15/16^fl/fl^) were deleted in Tregs after Foxp3 was expressed. By the age of 20 weeks, miR-15/16^fl/fl^*Foxp3*^Cre^ mice developed spontaneous systemic inflammation and lymphoproliferation (Figures 1A–1D). Specifically, we observed a significant infiltration of inflammatory cells in lung tissue, clusters of inflammatory cells in liver, and thickening of the skin, but no apparent inflammatory features in the pancreas (Figure 1A). Furthermore, spleen and inguinal lymph nodes were visibly larger compared with those of miR-15/16^wt/wt^*Foxp3*^Cre^ and miR-15/16^fl/fl^*Foxp3*^Wt^ control mice (hereafter referred to as “WT control”) (Figure 1B), and spleens obtained from miR-15/16^fl/fl^*Foxp3*^Cre^ mice had significantly greater mass (Figure 1C). The total number of cells was increased in spleen and lymph nodes of miR-15/16^fl/fl^*Foxp3*^Cre^ mice compared with WT control mice (Figure 1D).

Splenocytes exhibited increased abundance of a variety of inflammatory cells including B cells, CD4^+^ and CD8^+^ T cells, monocytes, eosinophils, and neutrophils (Figure 1E, T cell percentages in Figure 1F). Focusing on CD4^+^ T cells, miR-15/16 deficiency in the Treg compartment led to higher numbers of both Tregs (Figure 1G) and T effector (Teff) cells (Figures 1H and 1I), indicating that the absence of miR-15/16 in Tregs has secondary effects on the effector response. Indeed, compared with WT controls, miR-15/16^fl/fl^*Foxp3*^Cre^ mice had higher frequencies of IFN-γ- and IL-10-producing cells, but a lower frequency of IL-2-producing cells (Figures 1J and 1K). There were no differences in the proportion of IL-13- or IL-17-producing cells (Figures 1J and 1K). Taken together, our findings of systemic inflammation in multiple tissues and activation of effector cells suggest that miR-15/16 are essential for normal Treg functions that maintain immune homeostasis and prevent spontaneous inflammation.

### miR-15/16-expressing Tregs better suppress induced inflammation

To investigate the impact of miR-15/16 in Tregs under inflammatory conditions following an immunological trigger, we sensitized mice with a single intraperitoneal injection of ovalbumin (OVA) in alum (day 0) and challenged the airways with OVA on 3 consecutive days (days 7, 8, and 9) 1 week later (Figure 1L). Bronchoalveolar lavage (BAL) collected 24 h after the last OVA challenge showed a significantly higher frequency of airway-infiltrating eosinophils in miR-15/16^fl/fl^*Foxp3*^Cre^ mice compared with WT controls, despite similar numbers of neutrophils, macrophages, and CD4^+^ and CD8^+^ T cells (Figure 1M). The airway eosinophilia in miR-15/16^fl/fl^*Foxp3*^Cre^ mice was accompanied by an accumulation of airway-resident Tregs in the lung tissue and BAL (Figure 1N), indicating that miR-15/16-deficient Tregs are unable to suppress OVA-induced inflammation as effectively as WT Tregs.

### miR-15/16 selectively restrict Treg expansion

**Figure 2.**
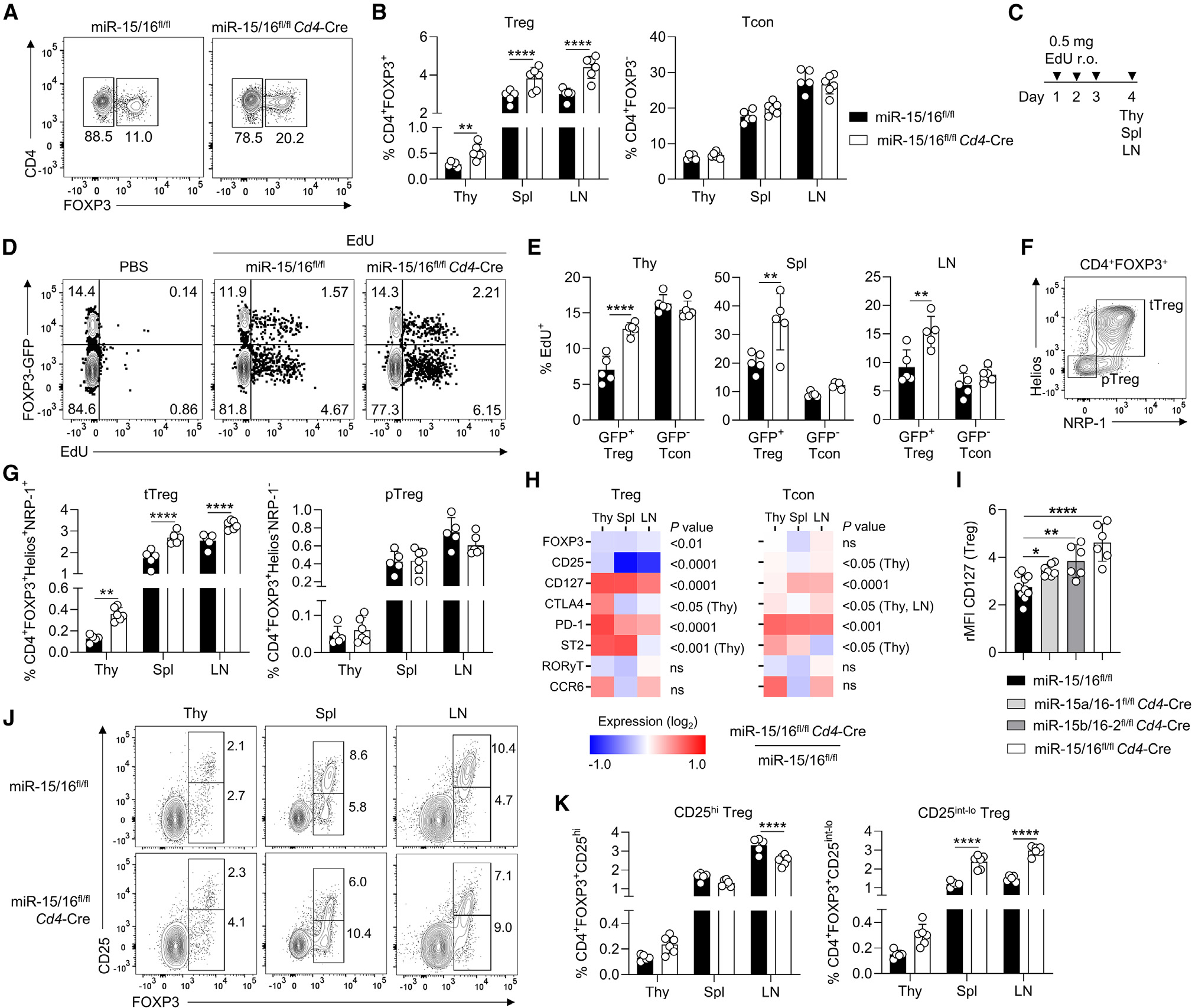
miR-15/16 specifically restrict the expansion of Tregs and regulate expression of key Treg proteins Representative contour plots (A) and quantification of frequencies (B) of CD4^+^FOXP3^+^ Tregs and CD4^+^FOXP3^−^ conventional T cells (Tcon) among live hematopoietic cells in thymus (Thy), spleen (Spl), and lymph nodes (LN) of miR-15/16^fl/fl^ WT control and miR-15/16^fl/fl^*Cd4*-Cre mice. (C) Model to track *in vivo* cell proliferation accomplished by retroorbital (r.o.) injection of 5-ethynyl-2′-deoxyuridine (EdU). Tissues were collected 24 h after the last injection. (D) Representative contour plots of EdU-injected miR-15/16^fl/fl^ WT control, miR-15/16^fl/fl^*Cd4*-Cre mice, and PBS control mice all carrying a FOXP3-GFP reporter. (E) Quantification of frequencies of CD4^+^GFP^+^ Tregs and CD4^+^GFP^−^ Tcons of all CD4^+^ cells in the indicated tissues of the same mice. (F) Contour plot demonstrating gating strategy of thymically derived tTregs (Helios^+^NRP-1^+^) and peripherally induced pTregs (Helios^−^NRP-1^−^). (G) Quantification of frequencies of tTregs and pTregs in the indicated tissues among live hematopoietic cells of miR-15/16^fl/fl^ WT control and miR-15/16^fl/fl^*Cd4*-Cre mice. (H) Fold change of median fluorescent intensity (MFI) by flow cytometry of the indicated proteins in Tregs and Tcons from three tissues (change in miR-15/16^fl/fl^*Cd4*-Cre from miR-15/16^fl/fl^ WT control). (I) Relative MFI (rMFI) of CD127 expression in Tregs from miR-15/16^fl/fl^ WT control, single cluster-deficient miR-15a/16–1^fl/fl^*Cd4*-Cre mice, miR-15b/16–2^fl/fl^*Cd4*-Cre mice, and double cluster-deficient miR-15/16^fl/fl^*Cd4*-Cre mice. Representative contour plots (J) and quantification of frequencies (K) of CD25^hi^ Tregs and CD25^lo^ Tregs among live hematopoietic cells in the indicated tissues of miR-15/16^fl/fl^ WT control and miR-15/16^fl/fl^*Cd4*-Cre mice. Data from a minimum of 2 independent experiments. N = 5–10 mice/group. In all bar graphs except (I), two-way ANOVA with Sidiak’s multiple comparison test. In (I) ordinary ANOVA with Dunnett’s multiple comparison test. Bar graphs are shown with error bars demonstrating standard deviation. Two-way ANOVA with Bonferroni’s multiple comparison test in heatmaps in (H). *p < 0.05, **p < 0.01, and ****p < 0.0001 for significance.

Our previous studies in *Cd4*-Cre mice, where miR-15/16 deficiency affects all T cells, demonstrate that miR-15/16 act through direct binding and posttranscriptional regulation of cell-cycle-associated genes to limit the expansion of CD4^+^ and CD8^+^ T cells during antiviral responses,^22^ but possible variations within the CD4^+^ T cell compartment were not investigated. Like the miR-15/16^fl/fl^ *Foxp3*^Cre^ mice, miR-15/16^fl/fl^*Cd4*-Cre mice exhibited an overgrowth of Tregs (Figures 2A and 2B). In fact, the increase in Tregs was selective as there was no difference in the number of FOXP3^−^ conventional T cells (Tcon) in miR-15/16^fl/fl^*Cd4*-Cre mice compared with miR-15/16^fl/fl^controls (Figure 2B). To examine whether the accumulation of Tregs was associated with heightened proliferation, we injected intravenous 5-ethynyl-2′-deoxyuridine (EdU) into miR-15/16^fl/fl^*Cd4*-Cre and control mice carrying a FOXP3-GFP reporter (Figures 2C and 2D). Among CD4^+^ T cells of the thymus, spleen, and lymph nodes, we detected increased EdU incorporation in GFP^+^ Tregs of miR-15/16^fl/fl^*Cd4*-Cre mice, but not in GFP^−^ Tcons (Figure 2E). We distinguished distinct Treg subsets of likely thymic (tTreg) or peripheral (pTreg) origin by their expression of Helios or NRP-1, respectively (Figure 2F).^30^ The increased Treg pool in miR-15/16^ffl/fl^*Cd4*-Cre mice consisted of tTregs, as there was no significant difference in the number of pTregs (Figure 2G). In summary, these findings suggest that miR-15/16 specifically restrict the accumulation of thymically derived Tregs, but not at the expense of conventional T cells or peripherally induced Tregs.

### miR-15/16 regulate the expression of key Treg proteins

In these experiments, we noted that Tregs that lack miR-15/16 display abnormally low FOXP3 expression (Figure 2A). Therefore, we quantified FOXP3 and other proteins critical to Treg function in both Tregs and Tcons from thymus, spleen, and lymph nodes (Figure 2H). FOXP3 and CD25 expression were substantially reduced in miR-15/16^fl/fl^*Cd4*-Cre Tregs compared with miR-15/16^fl/fl^ control Tregs (Figure 2H). In contrast, expression of the inhibitory receptors CTLA4 and PD-1 were enhanced in miR-15/16^fl/fl^*Cd4*-Cre Tregs (Figure 2H). Slight but significant effects were also seen in Tcons, where PD-1 expression was most clearly affected (Figure 2H). However, expression of proteins not directly implicated in bona fide Treg functions (ST2, RORγT, and CCR6) exhibited no or less consistent changes (Figure 2H). Not surprisingly, the direct miR-15/16 target CD127 was derepressed in the absence of miR-15/16 in both Tregs and Tcons (Figure 2H). Using mice lacking one or both of the two miR-15/16 clusters in T cells, we identified a dose-response of CD127 derepression with stepwise increases in CD127 abundance in a fashion related to the expression of the miRNAs in Tregs (Figure 2I). Further analysis of the impact of each miR-15/16 cluster on Treg cell number (*in vitro* and *in vivo*) and Treg phenotype is provided in Figure S2. These results show that miR-15/16-deficient Tregs have significant alterations in the expression of central functional proteins. FOXP3^lo^CD25^lo^ Tregs accumulated in all tissues that were investigated in miR-15/16^fl/fl^*Cd4*-Cre mice (Figures 2J and 2K). Together, the data suggest that miR-15/16 play an important role not only in controlling the size of the Treg pool but also in generating and maintaining a competitive Treg phenotype under homeostatic conditions.

### CD25^lo^ Tregs protect mice from intestinal inflammation

**Figure 3.**
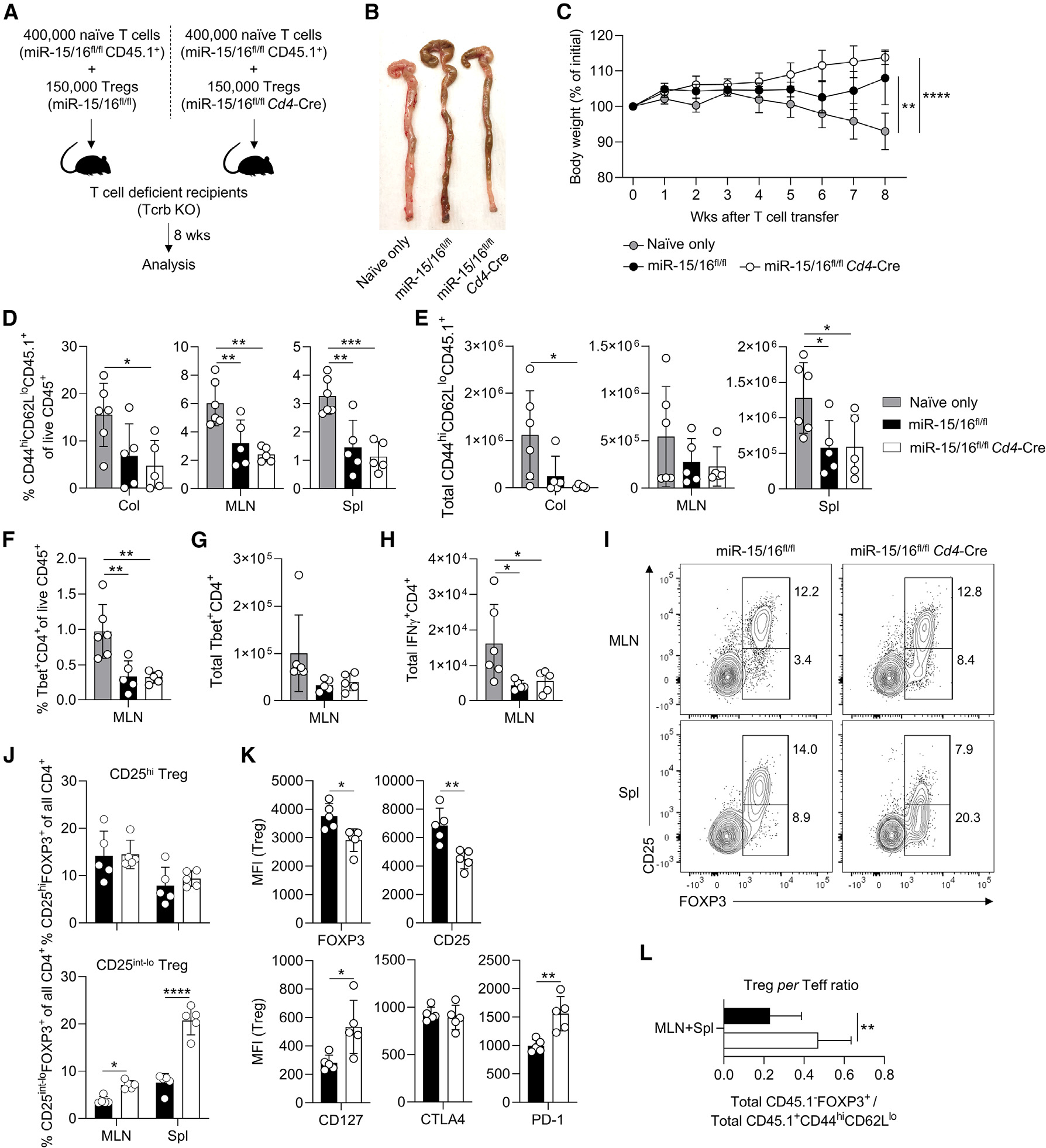
Accumulation of CD25^lo^ Tregs provides sufficient protection from intestinal inflammation (A) Colitis model by T cell transfer accomplished by intravenous injection of FACS-sorted naive T cells and Tregs. Recipient mice were monitored for 8 weeks before analysis. (B) Representative image of colons collected from mice that received naive T cells (“Naive only”), naive T cells together with miR-15/16-sufficient Tregs (“miR-15/16^fl/fl^”), and naive T cells together with miR-15/16-deficient Tregs (“miR-15/16^fl/fl^*Cd4*-Cre”). (C) Body weight monitored weekly of the same mice. Quantification of frequencies of CD44^hi^CD62L^lo^ T effector cells among live hematopoietic cells (D), and total CD44^hi^CD62L^lo^ T effector cells (E) in colon (Col), mesenteric lymph nodes (MLN), and spleen (Spl) of Naive only, miR-15/16^fl/fl^, and miR-15/16^fl/fl^*Cd4*-Cre-recipient mice. Quantification of frequencies of Tbet^+^CD4^+^ T cells among live hematopoietic cells (F) and total Tbet^+^CD4^+^ T cells (G) in MLN, and total IFN-γ^+^CD4^+^ T cells (H) in mesenteric lymph nodes of Naive only, miR-15/16^fl/fl^, and miR-15/16^fl/fl^*Cd4*-Cre-recipient mice. Representative contour plots (I) and quantification of frequencies (J) of CD25^hi^ and CD25^lo^ Tregs among all CD4^+^ T cells in mesenteric lymph nodes and spleen of miR-15/16^fl/fl^ and miR-15/16^fl/fl^*Cd4*-Cre-recipient mice. (K) Expression by median fluorescent intensity (MFI) using flow cytometry of the indicated proteins in spleen Tregs of miR-15/16^fl/fl^ and miR-15/16^fl/fl^*Cd4*-Cre-recipient mice. (L) Ratio of total number of FOXP3^+^ Tregs over total number of CD44^hi^CD62L^lo^ T effector cells (Teff) in mesenteric lymph nodes and spleen of miR-15/16^fl/fl^ and miR-15/16^fl/fl^*Cd4*-Cre Treg recipients. Data from 2 independent experiments. N = 5–6 mice/group. Ordinary ANOVA with Dunnett’s multiple comparison test in (D–H). Two-way ANOVA with Bonferroni’s multiple comparison test in (C and J), and unpaired t test two-tailed in (K and L). Graphs are shown with error bars demonstrating standard deviation. *p < 0.05, **p < 0.01, ***p < 0.001, and ****p < 0.0001 for significance.

To test the functional implications of miR-15/16 deficiency in Tregs, we gave T cell-deficient recipient mice 400,000 naive WT T cells together with 150,000 Tregs (CD4^+^CD45.1^−^FOXP3-GFP^+^) from either miR-15/16-sufficient mice (“miR-15/16^fl/fl^”) or miR-15/16-deficient mice (“miR-15/16^fl/fl^*Cd4*-Cre”) (Figure 3A). A third group received 400,000 naive WT T cells alone (CD4^+^CD45.1^+^CD25^−^CD44^lo^CD62L^hi^; “Naive only”) (Figure 3A). Recipient mice were monitored for wasting disease by body mass and condition over 8 weeks. Mice that received naïve T cells without Treg co-transfer developed colitis with visible inflammation of the colon (Figure 3B) and decreased body weight (Figure 3C). However, mice that received Tregs from either miR-15/16^fl/fl^ or miR-15/16^fl/fl^ *Cd4*-Cre mice had no visible inflammation of the colon (Figure 3B) or lower body weight by the end of the model (Figure 3C). The protective effect by Tregs was reflected by significantly lower frequencies and absolute numbers of Teff cells (CD4^+^CD44^hi^CD62L^lo^) in the colon, mesenteric lymph nodes, and spleen (Figures 3D and 3E). T cells expressing T-bet (Figures 3F and 3G) and IFN-γ (Figure 3H) were similarly reduced. The spontaneous accumulation of CD25^lo^ Tregs, previously found in mice lacking miR-15/16 expression in all T cells (miR-15/16^fl/fl^ *Cd4*-Cre) or in Tregs only (miR-15/16^fl/fl^*Foxp3*^Cre^), was replicated in recipient mice that obtained miR-15/16-deficient Tregs (Figures 3I and 3J). This result suggests that miR-15/16-dependent Treg expansion is not controlled by external factors, a question we address with greater detail in the next section. Frequencies of CD25^hi^ Tregs were found at the same level in miR-15/16^fl/fl^ and miR-15/16^fl/fl^*Cd4*-Cre Treg recipients (Figures 3I and 3J). Significantly reduced expression levels of FOXP3 and CD25, and enhanced expression of CD127 and PD-1 were characteristic of miR-15/16-deficient Tregs (Figure 3K), as reported in previous experiments. Accumulation of these abnormal Tregs resulted in higher Treg-to-Teff ratio in recipient mice (Figure 3L), possibly suggesting that Tregs lacking miR-15/16 expression compensate for impaired suppressive capacity and provide protection from intestinal inflammation by increasing in number.

### Accumulation of FOXP3^lo^CD25^lo^CD127^hi^ Tregs happens by cell-intrinsic mechanisms

**Figure 4.**
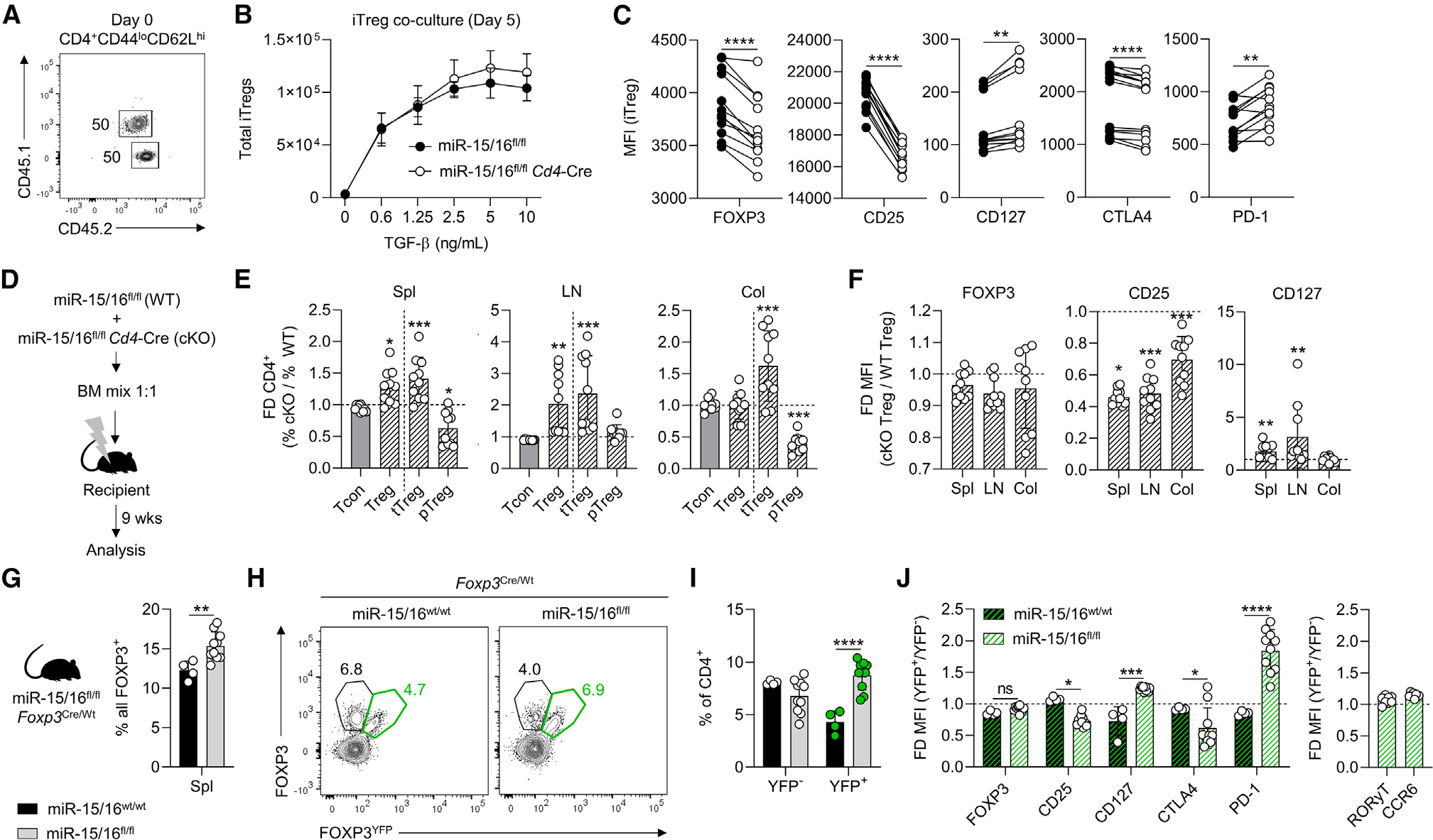
Accumulation of FOXP3^lo^CD25^lo^CD127^hi^ Tregs happens in a cell-intrinsic manner (A) Co-cultures of naive CD4^+^ T cells from CD45 congenic mice (miR-15/16^fl/fl^ and miR-15/16^fl/fl^*Cd4*-Cre). (B) Total number of induced Tregs (iTregs) after 5 days in culture under Treg polarizing conditions assessed by FOXP3 expression by flow cytometry. (C) Protein expression by median fluorescent intensity (MFI); paired analysis of co-cultured iTregs. (D–F) (D) Generation of mice with chimeric bone marrow. Recipient mice were analyzed after 9 weeks. Fold difference of cell abundance (E) and MFI by flow cytometry of the indicated proteins in Tregs (F) from three tissues (change in miR-15/16^fl/fl^*Cd4*-Cre from miR-15/16^fl/fl^ WT control). (G) Frequency of Tregs among all CD4^+^ T cells in natural chimeric female miR-15/16^fl/fl^*Foxp3*^Cre/Wt^ mice. (H) Representative contour plots of gating strategy to separate FOXP3^+^ cells based on YFP reporter signal (indicating Cre expression). YFP^−^ and YFP^+^ Treg frequency among all CD4^+^ T cells (I) and fold difference of flow cytometry MFI of the indicated proteins expressed in Tregs (change in YFP^+^ Tregs from YFP^−^ Tregs) (J). Data from a minimum of 2 independent experiments. N = 4–10 mice/group. Paired t test two-tailed in (C). Ordinary ANOVA with Dunnett’s multiple comparison test in (E and F). Unpaired t test two-tailed in (G), and two-way ANOVA with Sidiak’s multiple comparison test in (I and J). Graphs are shown with error bars demonstrating standard deviation. *p < 0.05, **p < 0.01, ***p < 0.001, and ****p < 0.0001 for significance.

Heightened expansion of miR-15/16-deficient Tregs in the colitis model indicated that the external environment of the host was not driving Treg accumulation and generation of an abnormal Treg phenotype. To study cell-intrinsic versus cell-extrinsic effects with greater detail, we co-cultured naive miR-15/16^fl/fl^ and miR-15/16^fl/fl^ *Cd4*-Cre T cells (CD4^+^CD44^lo^CD62L^hi^, distinguishable by the congenic marker CD45.1) under Treg-polarizing conditions (Figure 4A). The same number of FOXP3^+^-induced Tregs (iTregs) of either genotype accumulated after 5 days in culture(Figure 4B). However, comparisons of co-cultured iTregs showed consistent reduction of FOXP3, CD25, and CTLA4 expression in miR-15/16^fl/fl^*Cd4*-Cre iTregs compared with miR-15/16^fl/fl^ controls, and CD127 and PD-1 expression was consistently upregulated in miR-15/16-deficient iTregs (Figure 4C). These results confirmed that intrinsic signals drive the observed phenotypic changes *in vitro*.

We came to the same conclusion *in vivo* using mixed hematopoietic chimeras. We transferred an equal ratio of miR-15/16^fl/fl^ and miR-15/16^fl/fl^*Cd4*-Cre bone marrow cells to lethally irradiated recipient mice, and analyzed them after 9 weeks of reconstitution (Figure 4D). miR-15/16^fl/fl^*Cd4*-Cre Tregs were more abundant than miR-15/16^fl/fl^ control Tregs in spleen and lymph nodes (Figure 4E). Specifically, Helios^+^ tTregs were responsible for expanding the Treg pool. FOXP3^−^ Tcons were found at a 1:1 ratio in all tissues (Figure 4E). FOXP3 expression trended lower in miR-15/16^fl/fl^*Cd4*-Cre Tregs compared with miR-15/16^fl/fl^ Tregs from the same mice (Figure 4F, left panel), and the expression of CD25 was consistently and robustly reduced in miR-15/16-deficient Tregs (Figure 4F, middle panel). Conversely, CD127 expression was upregulated severalfold in spleen and lymph node Tregs lacking miR-15/16 (Figure 4F, right panel).

Finally, we used miR-15/16^fl/fl^*Foxp3*^Cre/Wt^ *Foxp3*-Cre-YFP heterozygous females as a third experimental approach that allowed us to study Cre-expressing miR-15/16-deficient Tregs (YFP^+^) and miR-15/16-sufficient Tregs (YFP^−^) in the same animal without intervention with irradiation and bone marrow transplantation. Despite allelic expression of Cre and YFP due to X chromosome inactivation, having only one *Foxp3*^Cre^ allele was enough to increase the total number of Tregs in female miR-15/16^fl/fl^*Foxp3*^Cre/Wt^ mice compared with miR-15/16^wt/wt^*Foxp3*^Cre/Wt^ controls (Figure 4G). Separating Tregs by YFP signal showed that these increases reflected a specific increase in the miR-15/16-deficient YFP^+^ Tregs in miR-15/16^fl/fl^*Foxp3*^Cre/Wt^ mice (Figures 4H and 4I), thereby confirming that miR-15/16 prevent Treg overgrowth in a cell-intrinsic manner. Moreover, these expanded miR-15/16-deficient Tregs also displayed the characteristic low expression of CD25 and CTLA4, and high expression of CD127 and PD-1 (Figure 4J). RORγT and CCR6 expression was similar in miR-15/16-deficient and -sufficient Tregs (Figure 4J). Together, these results demonstrate that miR-15/16 regulate gene expression networks in Tregs that limit their proliferation and modulate the expression of hallmark proteins.

### Access to IL-2 does not restore Treg CD25 expression in the absence of miR-15/16

**Figure 5.**
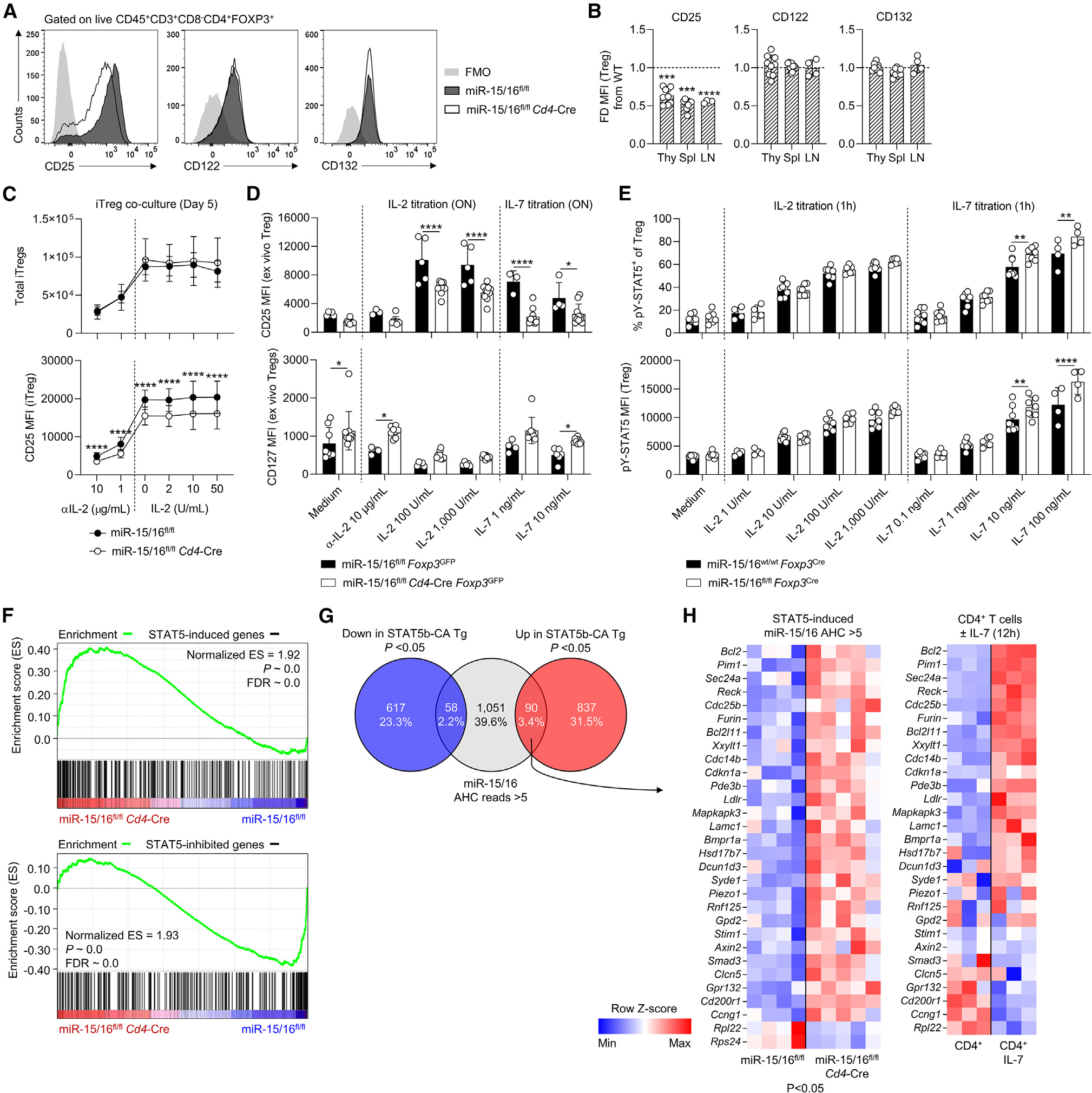
Derepression of miR-15/16 target CD127 promotes activation of STAT5 via IL-7 IL-2R subunit expression in representative flow cytometry histograms (A) and fold difference of expression by median fluorescent intensity (MFI) (B) in miR-15/16^fl/fl^ and miR-15/16^fl/fl^*Cd4*-Cre Tregs from the indicated tissues (change in miR-15/16^fl/fl^*Cd4*-Cre from miR-15/16^fl/fl^ WT control). (C) Total number of cells and CD25 expression by MFI in co-cultures of induced Tregs (iTregs) from CD45 congenic mice (miR-15/16^fl/fl^ and miR-15/16^fl/fl^*Cd4*-Cre) under different IL-2 concentrations. (D) CD25 and CD127 expression by MFI in Tregs isolated by FACS and cultured overnight (ON) with IL-2, IL-7, or in medium. (E) *Ex vivo* CD4^+^ T cell cultures from miR-15/16^wt/wt^ *Foxp3*^Cre^ and miR-15/16^fl/fl^*Foxp3*^Cre^ mice stimulated for 1 h with IL-2, IL-7, or unstimulated medium control followed by flow cytometry analysis of frequency of Tregs with phosphorylated STAT5 (anti-pY694-STAT5) and Treg pSTAT5 MFI. (F) Gene set enrichment analysis (GSEA) of genes upregulated (“STAT5-induced genes”) or downregulated (“STAT5-inhibited genes”) in Tregs with constitutively active STAT5b (extracted from Chinen et al.^6^) in CD4^+^ T cells from miR-15/16^fl/fl^ *Cd4*-Cre mice and miR-15/16^fl/fl^ control mice.^22^ (G) Venn diagram demonstrating overlap between STAT5-inhibited genes (“Down in STAT5b-CA Tg”), STAT5-induced genes (“Up in STAT5b-CA Tg”), and putative miR-15/16 target genes identified by Ago2 high-throughput sequencing of RNAs isolated by crosslinking immunoprecipitation (AHC) with read depth >5.^22^ (H) Expression of overlap genes (“miR-15/16 AHC reads >5” and “Up in STAT5b-CA Tg”) in CD4^+^ T cells of miR-15/16^fl/fl^ and miR-15/16^fl/fl^*Cd4*-Cre mice by RNA-seq,^22^ and their expression in CD4^+^ T cells after IL-7 stimulation.^55^ Data from a minimum of 2 independent experiments. N = 4–9/group. Ordinary ANOVA with Dunnett’s multiple comparison test in (B). Two-way ANOVA with Sidiak’s multiple comparison test in (C–E). Graphs are shown with error bars demonstrating standard deviation. *p < 0.05, **p < 0.01, ***p < 0.001, and ****p < 0.0001 for significance.

IL-2 signaling plays a fundamental role in Treg homeostasis and function, and exposure to IL-2 induces expression of its own high-affinity receptor chain, CD25 (also known as IL-2Rα).^6,31^ The other subunits of the IL-2R complex (CD122/IL-2Rβ and CD132/IL-2Rγ; also known as “common γ chain”) were expressed similarly in Tregs from miR-15/16^fl/fl^*Cd4*-Cre and miR-15/16^fl/fl^ control mice (Figures 5A and 5B). To test whether reduced IL-2 availability could be responsible for impaired CD25 expression in miR-15/16-deficient Tregs, we treated co-cultured iTregs from miR-15/16^fl/fl^ and miR-15/16^fl/fl^*Cd4*-Cre mice with different IL-2 concentrations including IL-2 neutralization by addition of an anti-IL-2 antibody. Neutralizing IL-2 reduced iTreg differentiation, affecting both genotypes similarly (Figure 5C). However, the amount of exogenously provided IL-2 did not alter the number of iTregs nor did it restore CD25 expression in miR-15/16-deficient iTregs (Figure 5C). In fact, miR-15/16-deficient iTregs responded to IL-2 stimulation by upregulating CD25 to the same degree as miR-15/16-sufficient iTregs (Figure 5C), although starting at a lower expression level prevented them from reaching full CD25 expression. *In vivo* generated Tregs sorted by FACS and cultured with or without IL-2 overnight confirmed this finding. CD25 expression increased in the presence of IL-2 in both control and miR-15/16-deficient Tregs (Figure 5D), with miR-15/16-deficient Tregs consistently exhibiting lower CD25 expression in all conditions (Figure 5D). These findings demonstrate that miR-15/16 are necessary to secure strong CD25 expression in Tregs independently of access to IL-2.

### Derepression of the miR-15/16 target CD127 promotes IL-7-dependent STAT5 activation

Tregs and Tcons display reciprocal expression of CD25 and CD127, with Tregs presenting as CD25^hi^CD127^lo^ and Tcons as CD25^lo^CD127^hi^. Consequently, IL-2 and IL-7 have opposing limiting functions in T cell immunity, with IL-2 required to maintain tolerance and IL-7 important for supporting immunological memory.^32^ Since miR-15/16 was required to maintain the CD25/CD127 balance in Tregs, we studied the effect of IL-7 in our *ex vivo* Treg cultures. IL-7 promoted T cell survival regardless of genotype, with little direct effect on CD25 and CD127 expression (Figure 5D). Next, we treated cells with IL-2 or IL-7 *ex vivo* and measured STAT5 phosphorylation (pSTAT5) in responding cells by flow cytometry. Despite the fact that miR-15/16 expression is required for high CD25 expression in Tregs, the frequency of pSTAT5^+^ cells and pSTAT5 signal intensity (MFI) were similar in miR-15/16-deficient and control Tregs in response to IL-2 stimulation (Figure 5E). In contrast, IL-7 stimulation selectively increased the frequency of pSTAT5^+^ cells and pSTAT5 MFI in miR-15/16-deficient Tregs, especially at high cytokine concentrations (Figure 5E).

IL-2R-deficient Treg function can be rescued by constitutive STAT5 expression.^6^ We extracted a list of differentially expressed gene (DEGs) by RNA sequencing (RNA-seq) of Tregs from transgenic STAT5b-CA mice and control Tregs and analyzed STAT5-induced or STAT5-inhibited genes in CD4^+^ T cells isolated from miR-15/16^fl/fl^*Cd4*-Cre and miR-15/16^fl/fl^ control mice. We identified a significant enrichment of STAT5-induced genes in miR-15/16^fl/fl^*Cd4*-Cre T cells, and an enrichment of STAT5-inhibited genes in miR-15/16^fl/fl^ control T cells (Figure 5F). Genes directly targeted by miR-15/16 in T cells were analyzed by Ago2 high-throughput sequencing of RNAs isolated by crosslinking immunoprecipitation (AHC) in our previous study.^22^ Putative miR-15/16 targets were identified as genes with one or more miR-15/16 3′ UTR seed matches corresponding with AHC read depth >5, and this gene set was highly enriched among genes upregulated in miR-15/16^fl/fl^*Cd4*-Cre T cells.^22^ Interestingly, this set of putative miR-15/16 targets overlapped with genes upregulated in Tregs with constitutive STAT5 expression (Figure 5G). Furthermore, several of these STAT5-induced putative miR-15/16 target genes were expressed at a significantly higher level in miR-15/16-deficient T cells compared with miR-15/16^fl/fl^ controls (Figure 5H), and a majority were induced in response to IL-7 stimulation (Figure 5H). Collectively, these findings suggest that increased IL-7R expression in miR-15/16-deficient Tregs may strengthen STAT5 signaling and the expression of downstream genes. In addition, many of those genes appear to be direct targets of miR-15/16, indicating that these miRNAs coordinate both cytokine responsiveness and cytokine-induced gene expression programs in Tregs.

### Treg overgrowth can compensate for impaired suppressive capacity

**Figure 6.**
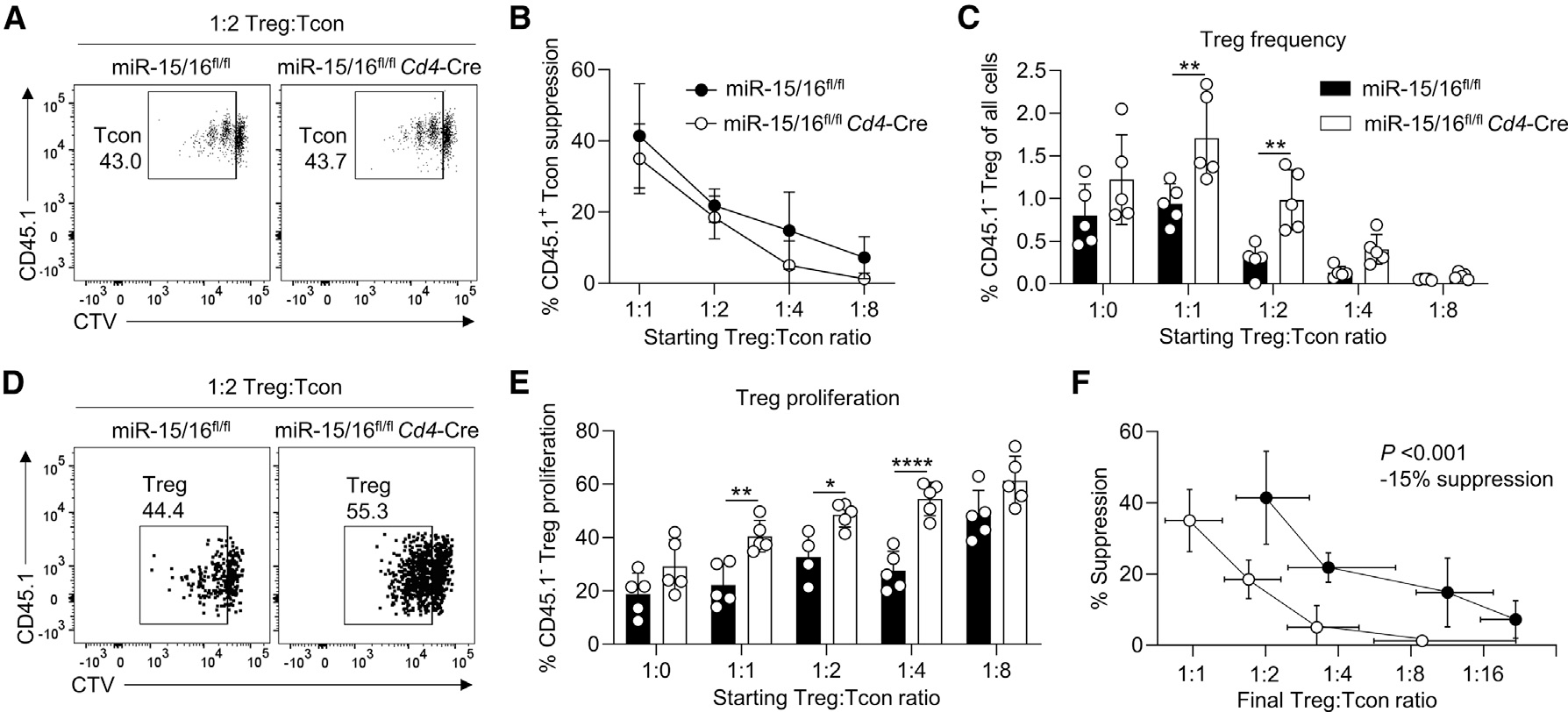
Treg overgrowth, caused by miR-15/16 deficiency, compensates for impaired suppressive ability (A) Representative dot plots of CellTrace Violet (CTV) dilution in CD4^+^CD25^−^CD45.1^+^ responder cells (Tcons) from WT mice, cultured with CD4^+^FOXP3-GFP^+^CD45.1^−^ Tregs from miR-15/16^fl/fl^*Cd4*-Cre *Foxp3*^GFP^ mice or miR-15/16^fl/fl^ *Foxp3*^GFP^ control mice. (B) Quantification of Treg suppression calculated based on Treg-to-Tcon seeding ratio at the start of culture. (C) Treg frequency among all T cells. (D) Representative dot plots of CTV dilution CD4^+^FOXP3-GFP^+^CD45.1^−^ Tregs from miR-15/16^fl/fl^*Cd4*-Cre *Foxp3*^GFP^ and miR-15/16^fl/fl^ *Foxp3*^GFP^ control. (E) Quantification of Treg proliferation (i.e., frequency of Tregs dividing ≥1). (F) Quantification of Treg suppression calculated based on Treg-to-Tcon seeding ratio at the end of culture (72 h post seeding). Data from a minimum of 2 independent experiments. N = 4–5/group. 2-way ANOVA with Sidiak’s multiple comparison test in (C) and (E), and linear regression analysis in (F). Graphs are shown with error bars demonstrating standard deviation. *p < 0.05, **p < 0.01, and ****p < 0.0001 for significance.

The inflammatory reactions happening systemically in miR-15/16^fl/fl^*Foxp3*^Cre^ mice indicate that Treg suppression is compromised in the absence miR-15/16, despite the increased number of Tregs in these mice. To measure the suppressive capacity of miR-15/16-deficient Tregs, we used *in vitro* suppression assays. CD4^+^CD25^−^CD45.1^+^ responder Tcons were sorted from WT mice and activated in presence of CD4^+^GFP^+^CD45.1^−^ Tregs sorted from miR-15/16^fl/fl^*Cd4*-Cre *Foxp3*^GFP^ and miR-15/16^fl/fl^*Foxp3*^GFP^ control mice. Surprisingly, Tcons cultured with miR-15/16^fl/fl^*Cd4*-Cre Tregs or miR-15/16^fl/fl^ control Tregs proliferated similarly, as indicated by CellTrace Violet (CTV) dye dilution (Figures 6A and 6B), suggesting that Tcons experienced the same degree of suppression by Tregs of either genotype. However, we analyzed Treg frequency 72 h after seeding the cells and found a significant increase in the number of miR-15/16^fl/fl^*Cd4*-Cre Tregs compared with control Tregs, which was enhanced in wells with responder cells present (Figure 6C). Labeling the Tregs with CTV confirmed increased proliferation in the absence of miR-15/16 (Figures 6D and 6E). We therefore recalculated the suppression profile, switching from the seeding ratio at the start of the culture to the final Treg-to-Tcon ratio at 72 h (Figure 6F). This analysis revealed that a greater number of Tregs from miR-15/16^fl/fl^*Cd4*-Cre mice were needed to achieve the same degree of suppression as Tregs from miR-15/16^fl/fl^ control mice. Using linear regression, we estimate 15% lower suppression by miR-15/16^fl/fl^*Cd4*-Cre Tregs (Figure 6F). These results demonstrate that miR-15/16 restrict Treg expansion *ex vivo*, and that excessive expansion due to miR-15/16 deficiency can compensate for lower suppressive capacity on a per-cell basis.

### miR-15/16-deficient Tregs resemble naturally occurring CD25^lo^ Tregs

**Figure 7.**
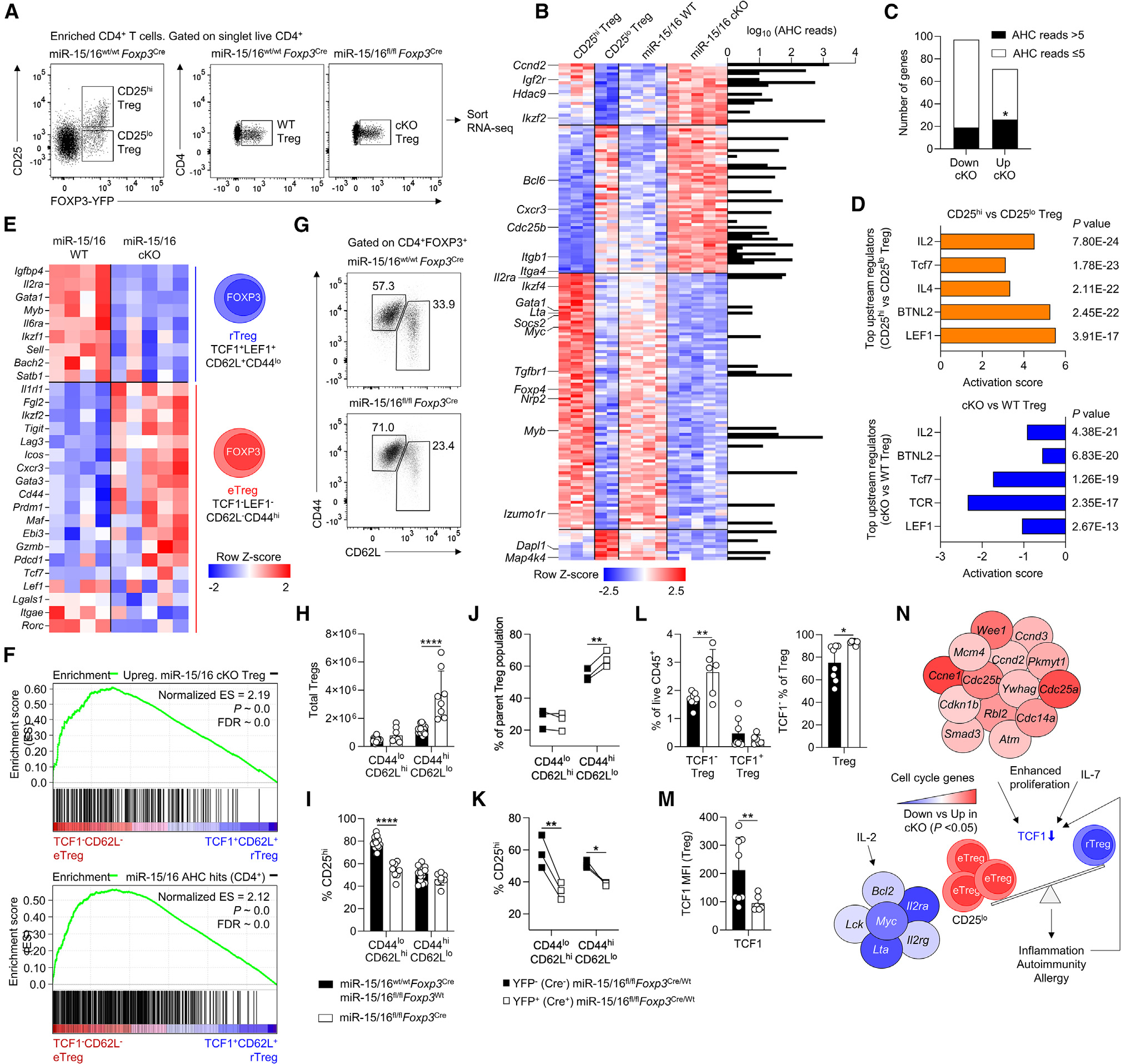
miR-15/16-mediated suppression of cell-cycle genes prevents downregulation of TCF1 and expansion of TCF1^−^ effector Tregs (A) Flow cytometry dot plots of Treg populations isolated for RNA-seq analysis (CD25^hi^ Tregs, CD25^lo^ Tregs, WT Tregs, and miR-15/16 cKO Tregs). (B) Heatmap of differentially expressed genes (DEGs) in CD25^hi^ versus CD25^lo^ Tregs and WT versus cKO Tregs (p < 0.05). The whole DEG list is provided as Table S1. The heatmap is plotted alongside a bar graph of AHC read depth at miR-15/16 seed matches for each gene at which they occur.^22^ (C) Genes with AHC reads >5 or AHC reads %5 at miR-15/16 seed matches that are downregulated and upregulated in cKO Tregs compared with WT Tregs.^22^ (D) Upstream regulators by ingenuity pathway analysis of DEGs in CD25^hi^ Tregs versus CD25^lo^ Tregs (top) and miR-15/16 cKO Tregs versus WT Tregs (bottom). (E) Heatmap expression of transcription factors and key functional molecules that distinguish resting Treg (rTreg) (TCF1^+^LEF1^+^CD62L^+^CD44^lo^) and effector Treg (eTreg) (TCF1^−^LEF1^−^CD62L^−^CD44^hi^) subgroups described by Yang et al.^11^ Expression in WT Tregs and miR-15/16 cKO Tregs is shown. (F) Gene set enrichment analysis (GSEA) of genes upregulated in cKO Tregs (top) (compared with WT) and AHC hits (reads >5) at miR-15/16 seed matches (bottom) across eTreg and rTreg transcriptomes.^11,22^ Representative dot plots (G) and quantification of total (H) CD62L^hi^CD44^lo^ Tregs and CD62L^lo^CD44^hi^ Tregs in miR-15/16^fl/fl^*Foxp3*^Cre^ mice and miR-15/16^wt/wt^ *Foxp3*^Cre^ and miR-15/16^fl/fl^ *Foxp3*^Wt^ control mice. (I) Frequency of CD25^hi^ cells among CD62L^hi^CD44^lo^ Tregs and CD62L^lo^CD44^hi^ Tregs in the same mice. (J) Frequency of CD62L^hi^CD44^lo^ and CD62L^lo^CD44^hi^ Tregs among all Tregs in heterozygous female miR-15/16^fl/fl^*Foxp3*^Cre/Wt^ mice and frequency of CD25-expressing cells among the same Treg populations in the same mice (K). Frequency of TCF1^−^ Tregs and TCF1^+^ Tregs among live hematopoietic cells (L, left), TCF1^−^ among Tregs (L, right), and TCF1 MFI in Tregs (M) in spleen of miR-15/16^fl/fl^*Foxp3*^Cre^ mice and miR-15/16^wt/wt^ *Foxp3*^Cre^ and miR-15/16^fl/fl^ *Foxp3*^Wt^ control mice. (N) Schematic illustration of proposed mechanism where enhanced proliferation due to increased cell-cycle gene expression leads to TCF1 downregulation to promote eTreg generation. eTregs, characterized by lower CD25 expression, have reduced IL-2-induced gene expression and insufficiently suppress immune activation. Data from a minimum of 2 independent experiments. N = 2–10 mice/group. Chi-squared test in (C). Two-way ANOVA with Sidiak’s multiple comparison test in (H and I). Paired t test two-tailed in (J and K), and unpaired t test two-tailed in (L and M). Bar graphs are shown with error bars demonstrating standard deviation. *p < 0.05, **p < 0.01, and ****p < 0.0001 for significance.

To learn more about the outcome of miR-15/16 deficiency in Tregs, we isolated Tregs from miR-15/16^fl/fl^*Foxp3*^Cre^ mice (“cKO Treg”) and miR-15/16^wt/wt^*Foxp3*^Cre^ controls (“WT Treg”) for RNA-seq analysis (Figure 7A). From miR-15/16^wt/wt^*Foxp3*^Cre^ mice, we also sorted CD25^hi^ and CD25^lo^ Tregs (Figure 7A). One hundred and sixty-eight genes were differentially expressed in CD25^hi^ versus CD25^lo^ Tregs and in WT versus cKO Tregs (Figure 7B; Table S1). DEGs included several known to modulate Treg function (e.g., *Myc*, *Myb*, *Bcl6*, *Tgfbr1*, *Ikzf2* [Helios], and *Ikzf4* [Eos]). Interestingly, the transcriptional profile of cKO Tregs resembled that of naturally occurring CD25^lo^ WT Tregs (Figure 7B). Mapping our AHC data onto the Treg DEGs revealed an enrichment for AHC reads corresponding with miR-15/16 seed sequences within the 3′ UTR of genes upregulated in cKO Tregs, compared with those more highly expressed in WT Tregs (Figure 7B, bars on right side). Quantifying this enrichment showed that significantly more genes upregulated in cKO Tregs had AHC reads ≥5 at miR-15/16 target sites (Figure 7C). Thus, miR-15/16 directly target some but not all of the DEGs shared by cKO Tregs and WT CD25^lo^ Tregs. A full transcriptome-wide analysis of cKO and WT Tregs demonstrated that miR-15/16 bind and regulate a large number of direct RNA targets in Tregs (Figure S3).

### miR-15/16 maintain a resting Treg transcriptional signature

We used Ingenuity Pathway Analysis to analyze all DEGs in CD25^hi^ versus CD25^lo^ Tregs to generate a list of candidate upstream regulators (Figure 7D). The same analysis performed on all DEGs in cKO versus WT Tregs identified several of the same upstream regulators, including *Il2*, *Tcf7* (TCF1), and *Lef1* (Figure 7D). Expression of the transcription factor TCF1 and its binding partner LEF1 was recently shown to separate Tregs into distinct subpopulations: activated Tregs, effector Tregs (eTregs), and resting Tregs (rTreg).^11^ Therefore, we analyzed the expression of transcription factors and key functional molecules that distinguish TCF1^+^LEF1^+^CD62L^+^CD44^lo^ rTregs from TCF1^−^LEF1^−^CD62L^−^CD44^hi^ eTregs in miR-15/16^fl/fl^*Foxp3*^Cre^ and miR-15/16^wt/wt^ *Foxp3*^Cre^ mice. The transcriptional profile of rTregs resembled that of WT Tregs, whereas eTregs corresponded more closely with cKO Tregs (Figure 7E). Expanding analysis to the entire transcriptome of rTregs and eTregs showed that genes upregulated in miR-15/16 cKO Tregs (Figure 7F, top) and miR-15/16 AHC hits (Figure 7F, bottom) were enriched in the eTreg transcriptional profile. Together, these results suggest that miR-15/16 maintain distinct subsets of Tregs with a resting phenotype.

### miR-15/16 restrict the formation of eTregs

In line with the eTreg transcriptional signature of miR-15/16 cKO Tregs, CD44^hi^CD62L^lo^ Tregs were selectively expanded in miR-15/16^fl/fl^*Foxp3*^Cre^ mice compared with miR-15/16^wt/w^t^^ *Foxp3*^Cre^ controls (Figures 7G and 7H). CD25 expression was markedly reduced among CD44^lo^CD62L^hi^ Tregs lacking miR-15/16, mimicking the lower frequency of CD25^hi^ cells in the CD44^hi^CD62L^lo^ Treg subset (Figure 7I). Analysis of the same Treg subsets in miR-15/16^fl/fl^*Foxp3*^Cre/Wt^ *Foxp3*-Cre-YFP heterozygous female mice confirmed that miR-15/16 deficiency promotes a selective increase of CD44^hi^CD62L^lo^ Tregs (Figure 7J) with reduced CD25 expression most pronounced in the CD44^lo^CD62L^hi^ Treg subset (Figure 7K). TCF1 protein expression was reduced in the whole Treg pool of miR-15/16^fl/fl^*Foxp3*^Cre^ mice, supporting a shift in transcriptional programs leading to expansion of TCF1^−^ eTregs (Figures 7L and 7M).

In summary, miR-15/16-deficient Tregs exhibited an effector Treg transcriptional signature and an increased proportion of cells with concordant expression of eTreg marker proteins. We propose a model wherein increased cell cycle and IL-7 signaling in the absence of miR-15/16 may be responsible for TCF1 downregulation that, in turn, promotes formation of eTregs that are characterized by lower CD25 expression, and hence decreased expression of IL-2-induced genes (Figure 7N). However, miR-15/16 affect a broad repertoire of direct targets in T cells, and other genes and pathways likely contribute to the observed Treg dysfunction. Our study demonstrates that miR-15/16 is essential for the high suppressive capacity of Tregs and elevated numbers of miR-15/16-deficient eTregs cannot prevent loss of tissue homeostasis, which results in immune activation and inflammation.

## DISCUSSION

Several decades of research have established Tregs as key suppressors of the immune system,^33^ where FOXP3 and IL-2 signaling via STAT5 orchestrate the core events that define and reinforce Treg identity.^6^ In more recent years, Treg heterogeneity has been explored,^11,34,35^ demonstrating that specific transcription factors, TCR repertoires, and cytokine signals drive functional specialization of Treg subsets.^11,36,37^ miRNAs exert their biologic effects through multiple targets in gene networks which makes them effective in regulating cellular trajectories.^22,38,39^ The experiments in this study revealed that miR-15/16 play a critical role in the regulation of Treg behavior. miR-15/16 expression was essential to develop a normal Treg phenotype under homeostatic conditions, to limit their proliferative capacity and efficiently suppress CD4^+^ Teff cells. Treg-specific ablation of miR-15/16 resulted in non-fatal but substantial systemic inflammatory responses in mice.

A recent study similarly identified that miR-15/16 play a key role in Treg biology^28^ but, in contrast to our study, the authors concluded that miR-15/16 deficiency enhanced Treg suppressive capacity. This independent work confirms our observation of selective accumulation of CD44^hi^ CD25^lo^ eTregs in the thymus and spleen of mice with Treg-specific miR-15/16 deficiency. However, there are several important differences between the studies. First, Dong et al.^28^ did not observe apparent signs of immune dysregulation, such as increased FOXP3^−^ Teff cell numbers in Treg-specific miR-15/16-deficient mice. This discrepancy may be explained in part by their focus on young mice (8–12 weeks). We did observe increased numbers of Teff cells in 8- to 11-week-old mice, but readouts of spontaneous inflammation were much more pronounced in 20-week-old mice. Second, different experimental systems were used to test Treg suppression *in vivo* (autoimmune neuroinflammation^28^ versus allergic lung inflammation). The differing outcomes in these settings indicate that the net effect of miR-15/16 on Treg suppression of inflammatory responses is context dependent. This was also highlighted by our observations in the colitis model and *in vitro* suppression assays, where increased Treg proliferation compensated for reduced suppressive function on a per-cell basis. In the future, it will be important to test whether Treg expansion can account for the improved suppression of neuroinflammation in Treg-specific miR-15/16-deficient mice.

It remains unclear why Tregs fail to suppress the immune system in chronic inflammatory diseases, and there is a need to increase our knowledge of the cellular programs capable of limiting inflammation and restoring tissue homeostasis. Experiments using OVA challenge of the airways to provoke type 2 inflammation elicited significantly more eosinophils in miR-15/16^fl/fl^*Foxp3*^Cre^ mice compared with WT controls, despite miR-15/16^fl/fl^*Foxp3*^Cre^ mice having a higher number of airway-resident Tregs. This disconnect between Treg numbers and physiological function highlights the importance of functional specialization in Treg suppression of immune activation. Spontaneous tissue inflammation prior to allergen challenge may have affected the ability of Tregs to suppress additional inflammatory cues induced by OVA in miR-15/16^fl/fl^*Foxp3*^Cre^ mice. Treg exhaustion has frequently been reported in malignancies where checkpoint blockade through PD-1 is an effective tumor treatment.^40^ miR-15/16^fl/fl^*Foxp3*^Cre^ Tregs were characterized by high PD-1 expression, a feature associated with exhaustion, IFN-γ production, and reduced suppression of CD4^+^ Teff cells.^41^ miR-15/16^fl/fl^*Foxp3*^Cre^ mice displayed increased numbers of Teff cells and elevated IFN-γ production, indicating possible Treg dysfunction related to cell exhaustion.

Based on prior research from the cancer literature, we speculate that direct miR-15/16 targeting of cell-cycle genes contributes to the enhanced proliferation of Tregs. Indeed, miR-15/16 are known tumor suppressors, best characterized in B cell leukemia, which affect cell growth by restricting cell-cycle and anti-apoptotic genes such as *Ccnd1* (cyclin D1), *Bmi1, Bcl2*, and *Mcl1*.^21,42^ Our previous work demonstrated that miR-15/16 also regulate CD8^+^ T cell expansion and differentiation through a large network of genes that control cell cycle, memory, and survival.^22^ This study revealed a discrepancy in the CD4^+^ T helper cell compartment, where miR-15/16 restrict expansion of Tregs but not Tcons. Among human T cells, Tregs express markedly higher miR-15/16 than do conventional Th1, Th2, Th17, and naive T cells. This observation may indicate more substantial dependence on miR-15/16 regulation in Tregs and is supported by a report of enhanced pTreg generation from miR-15/16-overexpressing conventional CD4^+^ T cells *in vivo*^27^ and miR-15/16 expression levels in murine Tregs and Tcons.^28^ Furthermore, we showed that miR-15/16 modulated expression of key Treg functional proteins. Throughout the study, miR-15/16-deficient Tregs were characterized as FOXP3^lo^CD25^lo^CD127^hi^ with lower CTLA4 and higher PD-1 expression compared with miR-15/16-sufficient cells in both lymphoid and non-lymphoid tissues (Figures S4A and S4B), a phenotype established by cell-intrinsic regulation. More subtle changes in protein expression were observed in Tcons obtained from miR-15/16^fl/fl^*Cd4*-Cre mice; however, secondary effects on the Tcon phenotype cannot be ruled out since Treg function was altered in those mice. Nevertheless, expression of the direct miR-15/16 target CD127 was altered in Tcons. While CD127 increased dramatically in miR-15/16-deficient Tregs, a subtle increase was observed in Tcons, possibly reflecting lower miR-15/16 activity in the latter. Activated CD4^+^ Tcons in the airways of OVA-challenged *Cd4*-cre mice showed differential expression of CD25, CD127, and PD-1, which demonstrates a dependence of miR-15/16 regulation in non-Tregs (Figure S4C). CD127 stimulation, via IL-7 or TSLP, is essential for Treg development,^43^ and IL-7 stimulation has been shown to temporarily rescue survival of Tregs after inducible CD25 ablation *in vivo*.^44^ In our study, CD127 upregulation in miR-15/16-deficient Tregs may have contributed to enhanced survival, while compensating for loss in IL-2-dependent survival due to impaired CD25 expression.

miR-15/16 regulation of Treg expansion was selective to tTregs, not pTregs, despite the subsets sharing a functional FOXP3^lo^CD25^lo^CD127^hi^ phenotype (Figure S4B). Similarly, *in vitro* generated iTregs did not exhibit excessive expansion in the absence of miR-15/16. CD25 signaling is essential for early expansion of tTreg and Treg homeostasis in the periphery, and CD25 cKO tTregs (*Foxp3*^Cre^) exhibit reduced levels of most Treg functional molecules despite nearly normal levels of FOXP3.^7^ These findings suggest that STAT5 regulation of FOXP3 expression in the thymus is largely IL-2 independent, consistent with previous reports of redundant STAT5 phosphorylation in developing Tregs by other STAT5-activating cytokines such as IL-7 and IL-15.^43,45,46^ In our study, enhanced Treg expression of CD127 may have promoted development and expansion of the tTreg pool. IL-7 stimulation resulted in increased pSTAT5^+^ cells in miR-15/16-deficient CD127^hi^ Tregs, and IL-7-responsive STAT5-induced genes overlapped with miR-15/16-regulated gene sets in CD4^+^ T cells.^22^ Interestingly, STAT5a binds near the miR-15b/16–2 cluster and suppresses miR-15b/16–2 expression,^47^ suggesting that miR-15/16 may participate in a feedforward circuit that enhances STAT5 transcriptional responses.

One of the most striking observations in our study is that miR-15/16 secure high CD25 expression in Tregs. IL-2 stimulation of *ex-vivo*-cultured Tregs confirmed a functionally responsive *Il2ra* locus with expression dynamics similar to those of WT Tregs, and CD25 function was further confirmed by normal generation of pSTAT5^+^ cells upon IL-2 stimulation. Nevertheless, miR-15/16-deficient Tregs exhibited changes in the expression of genes that distinguish naturally occurring CD25^lo^ Tregs in WT mice. CD25 expression in Tregs declines progressively with age and is associated with development of autoimmune diseases and immune senescence.^42,48,49^ Thus, Treg-specific miR-15/16 deficiency may be a useful model for age-related inflammation. The mechanisms that underlie reduced CD25 expression in Tregs are unclear, but a recent comprehensive CRISPR screen identified several transcription factors that negatively regulate CD25 expression in human CD4^+^ T cells.^50^ Reduced IL-2 production has been suggested to promote lower Treg CD25 expression^44^; however, it does not explain the CD25^lo^ phenotype of miR-15/16-deficient Tregs. Our data from chimeric mice, adoptive transfer, and co-culture experiments demonstrate a Treg-intrinsic effect by miR-15/16 on CD25 expression.

In our transcriptional analysis, the striking overlap of upstream regulators in miR-15/16-deficient Tregs and CD25^lo^ Tregs from WT mice revealed a strong association of TCF1 with miR-15/16 gene networks and CD25 signaling. Other reports also suggest close integration of these pathways, for example, cell-cycle progression is regulated by miR-15/16, upregulated in CD25-deficient Tregs,^7^ and promotes generation of TCF1^−^ cells.^51^ In addition, IL-7 signaling inhibits TCF1 expression in thymocytes and mature T cells,^52^ and TCF1 deficiency in mice leads to increased thymic output of Tregs.^53^ While TCF1^−^LEF1^−^ Tregs exhibit active cell cycle and increased proliferation, their suppressive capacity has been reported to remain intact.^11^ In this study, we observed situations where miR-15/16 exerted compensatory effects on Treg control of CD4^+^ T cell responses. For instance, miR-15/16-deficient Tregs proved capable of suppressing Tcon proliferation *in vitro*, but closer inspection revealed that increased Treg proliferation compensated for lower suppressive capacity on a per-cell basis. The same situation appeared in the *in vivo* cell transfer system, where miR-15/16-deficient Tregs protected mice from intestinal inflammation, while also expanding to greater numbers compared with WT Tregs. Furthermore, miR-15/16 depletion in all T cells did not result in inflammation and autoimmune disease in *Cd4*-Cre mice, but conditional depletion of miR-15/16 in Tregs did. The onset of autoimmune disease in *Foxp3*^Cre^ mice suggests that the shift in the Treg pool to a higher proportion eTregs was not sufficient to keep autoimmunity in check, and our data support previous reports that demonstrate the importance of having functionally diverse Treg subsets to prevent disease.^11^

We conclude that miR-15/16 represents a critical node in the coordinate regulation of the heterogeneity, expansion, and suppressive function of the Treg pool. Reducing miR-15/16 expression might be beneficial in cancer immunotherapies where decreased IL-2 sensitivity in Tregs could limit IL-2-induced Treg amplification while maintaining IL-2 responsiveness in tumor-infiltrating lymphocytes. Further investigation of miR-15/16 regulation of Tregs may uncover therapeutic targets to modulate Treg suppression in the setting of cancer or autoimmunity.

### Limitations of the study

Further work is required to clarify the mechanism(s) by which miR-15/16 regulate Treg responses. We used gene expression profiling to characterize the effects of miR-15/16 deficiency in Tregs, but those data reflect the direct effects of miR-15/16 repression of mRNA targets, indirect effects downstream of those direct targets, and possibly even more indirect, cell-extrinsic effects related to global Treg dysfunction and the resulting breakdown in immune tolerance. It may be possible to resolve some of these complexities by comparing data from this study with recently generated transcriptional profiles of miR-15/16-sufficient and -deficient Tregs from heterozygous miR-15/16^fl/fl^*Foxp3*^Cre/Wt^ female mice.^28^ Another limitation of our study is that putative miR-15/16 targets were extracted from AHC experiments conducted in miR-15/16^fl/fl^*Cd4*-Cre T cells,^22^ raising the possibility that less-abundant Treg-specific miR-15/16 targets could be overlooked. Some of the very few mRNAs expressed very selectively in Tregs (e.g., *Foxp3*) could bind miR-15/16 and be missed in our current analysis. It is also formally possible that there could be accessibility effects that generate cell-type-specific binding, although prior research has indicated that such cases are unusual. To fully decipher miR-15/16 regulatory networks in Tregs, comparative AHC experiments using Tregs with and without miR-15/16 expression should be used to definitively map binding sites in the Treg transcriptome.

## STAR★METHODS

### RESOURCE AVAILABILITY

#### Lead contact

Further information and requests for resources and reagents should be directed to and will be fulfilled by the Lead Contact K. Mark Ansel (mark.ansel@ucsf.edu).

#### Materials availability

This study did not generate new unique reagents.

#### Data and code availability

- Raw RNA-seq data and processed files reported in this paper are available on NCBI GEO with accession number GEO: GSE230099. miRNA expression data by RT-PCR in sorted human blood T cell populations was generated by Rossi et al.^29^ (NCBI GEO accession number GEO: GSE22880). RNA-seq data of Tregs with constitutive STAT5b expression was generated by Chinen et al.^6^ (GEO: GSE84553), and RNA-seq data of IL-7 stimulated mouse CD4^+^ T cells was generated by Villarino et al.^55^ ( GEO: GSE77656). Finally, RNA-seq data of miR-15/16-deficient and sufficient CD4^+^ T cells and AHC were generated by Gagnon et al.^22^ (GEO: GSE111568), and RNA-seq data of TCF1^−^LEF1^−^ Tregs was generated by Yang et al.^11^ (GEO: GSE117726).
- This paper does not report original code.
- Any additional information required to reanalyze the data reported in this work paper is available from the lead contact upon request.

### EXPERIMENTAL MODEL AND STUDY PARTICIPANT DETAILS

#### Mice

miR-15/16^fl/fl^*Cd4*-Cre mice with conditional inactivation of miR-15/16 family miRNAs (miR-15a, miR-15b, miR-16–1, miR-16–2) in T cells, inactivation of single miR-15a/16–1 or miR-15b/16–2 clusters in T cells, and mice for hematopoietic chimeras were generated as described previously.^22^ miR-15/16^fl/fl^*Foxp3*^Cre^ mice with conditional inactivation of miR-15/16 family miRNAs in Tregs were generated by crossing miR-15/16^fl/fl^ mice (loxP-flanked miR-15a/16–1 and miR-15b/16–2 alleles^22^) with B6.129(Cg)-*Foxp3*^*tm4(YFP/icre)Ayr*^/J mice, kindly provided by the Rosenblum lab (University of California San Francisco). miR-15/16^fl/fl^*Cd4*-Cre mice carrying a FOXP3 GFP-reporter were generated by crossing miR-15/16^fl/fl^*Cd4*-Cre mice^22^ with B6.Cg-*Foxp3*^*tm2Tch*^/J mice. In all experiments, male and female age and sex matched mice were used between 8 and 11 week of age or at 20 weeks of age as stated in the text. All mice were housed and bred in specific pathogen-free conditions in the Animal Barrier Facility at the University of California San Francisco. Animal experiments were approved by the Institutional Animal Care and Use Committee of the University of California San Francisco.

### METHOD DETAILS

#### OVA model

The OVA model is outlined in Figure 1K. Mice were injected intraperitoneally with 50 μg OVA (Sigma-Aldrich) resuspended in 100 μL of sterile PBS and mixed with 100 μL of Imject alum (Thermo Fisher Scientific). After 7 days, the mice were challenged on three consecutive days by intranasal administration of 50 μg OVA resuspended in 20 μL of sterile PBS. Control mice received intranasal doses of 20 μL sterile PBS. 24 h after the last intranasal challenge, the mice were anesthetized with isoflurane and 2 μg anti-CD45-BV510 antibody was injected by retroorbital route to label circulating blood cells. The mice were sacrificed 2–3 min later to collect BAL fluid and lung tissue. BAL was performed by 4 consecutive washes with 0.25 mL sterile PBS. BAL cells were incubated in ACK buffer (Lonza) for 2 min at RT to remove contaminating red blood cells, washed, resuspended in FACS buffer (2% FBS in PBS) and stored on ice until flow cytometry staining (described below). Airway inflammation was assessed by analysis of inflammatory cells in the BAL (eosinophils, neutrophils, alveolar macrophages, CD4^+^ T cells and CD8^+^ T cells), and frequency of Tregs was also measured in BAL. After BAL collection, lungs were PBS perfused by heart injection and left and right lung lobes were collected. Lung tissue was transferred to gentleMACS C-tubes (Miltenyi Biotec) containing 5 mL of freshly prepared lung digestion medium: 12.5 μg/mL DNase I (Sigma-Aldrich), 1.2 mg/mL Collagenase D (Roche) in RPMI 1640 (Fisher Scientific). The tissue was disrupted by running program m_lung_01 on the gentleMACS dissociator (Miltenyi Biotec) followed by 30 min incubation at 37°C with continuous shaking. Next, program m_lung_02 was used on the the gentleMACS dissociator and the tissue suspension was filtered through 70 μm Corning cell strainers (Fisher Scientific). Cells were incubated in ACK buffer for 2 min at RT, washed, resuspended in FACS buffer and stored on ice until flow cytometry staining of tissue-resident Tregs.

#### *In vivo* proliferation by EdU

For EdU labeling of proliferating cells, 0.5 mg EdU in 200 μL sterile PBS was injected retroorbitally on three consecutive days before sacrifice, 24 h after the last injection (Figure 2C). Thymus, spleen and inguinal lymph nodes were collected and cell suspensions were prepared by passing the tissues through 70 μm cell strainers. Spleen cells were incubated in ACK buffer for 2 min at RT. All cells were resuspended in FACS buffer and stored on ice until flow cytometry staining of EdU^+^ cells using the Click-iT Plus EdU Flow cytometry assay kit according to the manufacturer’s instructions (Thermo Fisher Scienticific).

#### Treg transfer model

The colitis model by T cell transfer is outlined in Figure 3A. T cells were enriched from spleen and lymph nodes of CD45.1^+^ mice by negative selection using EasySep Mouse CD4^+^ T cell isolation kit (STEMCELL Technologies), and FACS sorted as CD4^+^CD25^−^CD44^lo^CD62L^hi^ cells. 400,000 naive T cells were injected retroorbitally together with 150,000 Tregs, FACS sorted as CD4^+^FOXP3-GFP^+^ from spleen and lymph nodes. The Tregs were obtained from CD45.1^−^ miR-15/16^fl/fl^*Cd4*-Cre mice or miR-15/16^fl/fl^ control mice. One group of mice received only 400,000 naive T cells without Treg co-transfer. The weight of Tcrb KO recipient mice was recorded one time per week for 8 weeks. By the end of the model, colon, mesenteric lymph nodes and spleen were collected for flow cytometric analysis of Teff and Treg frequencies, and cytokine expression. Cell suspensions from spleen and lymph nodes were generated as described above. Colon was flushed with 10 mL of sterile PBS, cut in half longitudinally, then cut into small pieces and placed in a Petri dish with 10 mL HEPES buffer: 15 mM HEPES (Fisher Scientific) in HBSS without calcium, magnesium, and phenol red (Fisher Scientific). The tissue pieces were gently agitated in the dish and washed in fresh HEPES buffer three times before being transferred to 10 mL 37°C digestion solution that consisted of RPMI-1640 supplemented with 2.5 mg/mL Collagenase D. Samples were incubated at 37°C with continuous shaking, and after 1h, the tissue suspension was filtered through 70 μm cell strainers. Remaining tissue pieces on the filter were placed in fresh digestion solution and incubated 1h at 37°C with continuous shaking. The procedure was repeated three times leaving no visible tissue pieces. Supernatants were centrifuged and cells were washed and resuspended in FACS buffer, pooling cells from all rounds of digestion.

#### Flow cytometric analysis

Single cell suspensions from thymus, spleen, lymph nodes, colon, and lungs were transferred to 96-well conical bottom plates (Thermo Fisher Scientific) for antibody staining. Cells were washed one time in plain PBS followed by incubation for 15 min at 4°C in fixable viability dye at 1:2000 dilution in PBS. FACS buffer was used to wash, and the cells were blocked by adding anti-CD16/CD32 antibodies at 1:100 dilution in FACS buffer and incubated for 15 min at 4°C. Antibodies for surface stain was added at 1:100 dilution in blocking solution and incubated for 30 min at 4°C (dark). FACS buffer was used to wash the cells followed by fixation for 20 min at RT using Foxp3/Transcription Factor Staining Buffer set (Thermo Fisher Scientific). Surface-stained cells were washed, 5,000 AccuCount beads (Spherotech) were added, and samples were acquired on a BD LSRII flow cytometer (BD Biosciences). For samples that were stained intracellularly, fixation was followed by wash in permeabilization buffer (Foxp3/Transcription Factor Staining Buffer set). Intracellular antibodies were added at 1:100 dilution in permeabilization buffer and incubated for 1h at 4°C (dark). Samples were washed two times, 5,000 AccuCount beads were added, and samples were acquired on a BD LSRFortessa cell analyzer (BD Biosciences). In experiments combining anti-FOXP3 antibody stain with endogenous FOXP3-YFP reporter, Foxp3/Transcription Factor Staining Buffer set was used as described above. Cells from lungs (OVA model) and mesenteric lymph nodes (colitis model) were re-stimulated *ex vivo* for cytokine staining and analysis by flow cytometry. In those experiments, cells were stimulated with 20 nM PMA and 1 μM ionomycin for 4h, where 5 μM brefeldin A was added during the final 2h. Following surface stain (described above), cells were fixed in 4% PFA for 8 min at RT. Ice-cold plain PBS was added and cells were washed one time in cold plain PBS and one time in SAP permeabilization buffer (0.2% saponin in FACS buffer). Antibodies for intracellular cytokine stain was diluted at 1:20 concentration in SAP permeabilization buffer and added to the cells for 1h incubation at 4°C (dark). Cells were washed in FACS buffer and followed by acquisition on the BD LSRFortessa cell analyzer. FlowJo V10 was used for the data analysis.

#### *In vitro* Treg polarization

Naive CD4^+^ T cells from spleen and lymph nodes were enriched by negative selection using EasySep Mouse Naive CD4^+^ T cell isolation kit (STEMCELL Technologies). 100,000 cells/well (50,000 CD45.1^−^ cells and 50,000 CD45.1^+^ cells) were added to 96-well plates (Costar 3370, Corning) coated with anti-CD3 (1 μg/mL) and anti-CD28 (0.5 μg/mL) antibodies. In IL-2 titration experiments, the cell culture medium was supplemented with 5 ng/mL recombinant human TGF-β (Peprotech), 10 μg/mL anti-IL-4 antibodies (clone 11B11), and 10 μg/mL anti-IFN-γ antibodies (clone XMG1.2) at seeding, and recombinant IL-2 (National Cancer Institute; concentrations indicated in figures) was added three days later when cells were moved to U-bottom 96-well plates with fresh culture medium. In TGF-β titration experiments, the cell culture medium was supplemented with TGF-β (as indicated in figures) at seeding along with anti-IL-4 and anti-IFN-γ antibodies at concentrations described above, and 20 units/ml IL-2 was added three days later. Cells were incubated at 37°C with 10% CO_2_ and iTregs were analyzed after 5 days in culture by flow cytometry.

#### *Ex vivo* Treg cultures

CD4^+^ T cells were enriched from spleen and lymph nodes by magnetic separation and surface stained as described above. Cells were filtered and resuspended in sorting buffer (FACS buffer supplemented with 2 mM EDTA). Tregs were sorted as singlet live CD4^+^FOXP3-GFP^+^ cells on a BD FACSAria II cell sorter. 100,000 Tregs/well were seeded in U-bottom 96-well plates, supplemented with recombinant IL-2 or IL-7 at concentrations indicated in figures, and incubated at 37°C with 10% CO_2_ ON. Cells were analyzed by flow cytometry the next day. For pSTAT5-staining, 500,000 CD4^+^ T cells enriched from spleen and lymph nodes were seeded in plain DMEM high glucose media and added to U-bottom 96-well plates. Cells were rested for 1h at 37°C with 10% CO_2_ before adding recombinant IL-2 or IL-7 in plain DMEM as indicated in figures. Cells were incubated 1h at 37°C with 10% CO_2_ and immediately fixed by adding 37°C 2% PFA followed by incubation for 10 min at RT. Cells were washed two times in FACS buffer then permeabilized with −20°C 100% methanol and stored ON in −20°C freezer. The next day, cells were washed two times in FACS buffer followed by rehydration for 20 min at RT in FACS buffer. Cells were washed and blocked as described above and incubated 30 min at RT (dark) with anti-STAT5 (pY694) antibodies (20 μL/test) and antibodies for surface stain (1:100 dilution). Cells were washed in FACS buffer and acquired on a BD LSRII flow cytometer where the endogenous FOXP3-YFP reporter was used to distinguish Tregs from other CD4^+^ T cells.

#### *In vitro* suppression assay

CD45.1^−^ Tregs were FACS sorted as described above, and CD45.1^+^ Tcons were sorted as singlet live CD4^+^FOXP3-GFP^−^CD25^lo^ cells. Tregs and Tcons were CTV-labeled (5 μM in PBS) by incubation for 20 min at 37°C. Cells were washed and resuspended in complete cell medium.^38^ Splenocytes from Tcrb KO mice were resuspended in 50 μg/mL Mitomycin C (Roche) in PBS and incubated at 37°C for 1h (mixed repeatedly), followed by three repeated washes in plain PBS. Each well was supplemented with 5 μg/mL anti-CD3 (clone 145–2C11), 100,000 Mitomycin C-treated splenocytes and 50,000 CTV-labeled CD45.1^+^ Tcons. Tregs and Tcons were cultured at different seeding ratios, starting at 1:1 Treg:Tcon ratio, followed by 2-fold Treg dilution to have 2×, 4×, and 8× more Tcons than Tregs. The cells were incubated for 72 h at 37°C with 10% CO_2_ before flow cytometric analysis of proliferation by CTV dilution.

#### Histology

Pancreas, the left lung lobe and the right liver lobe were placed in 10% formalin for 48h before transfer to PBS for storage. Skin samples were collected from the neck and placed in formalin for 5h then transferred to 30% sucrose for 48h followed by storage in PBS. Tissues were sectioned and H&E stained according to standard protocol.

#### RNA sequencing and bioinformatic analysis

Tregs (bulk cKO, bulk WT and WT CD25^hi^ and CD25^lo^ bin) were sorted by FACS as described above using the endogenous FOXP3-YFP reporter. 340,000–1,200,000 cells were lysed in QIAzol (Qiagen) and total RNA was isolated using miRNeasy Mini Kit (Qiagen) with on-column DNase digestion. RNA quality and yield was determined on an Agilent 2100 Bioanalyzer (Agilent BioTek). cDNA was synthesized using the Nugen Universal Plus library kit (NuGen Technologies). The samples were sequenced using single-end 50 bp RNAseq on the Illumina HiSeq 4000 platform. Alignment was performed using STAR_2.7.2b against the Ensembl Human GRCm38.78 alignment genome. Differential expression was tested using DESeq2, and expression values were analyzed as log2 fold change of miR-15/16 deficient Tregs compared to miR-15/16 sufficient Tregs, or CD25^hi^ Tregs compared to CD25^lo^ Tregs.

Ingenuity Pathway Analysis (IPA; Qiagen) was used to identify upstream regulators of gene expression networks in Tregs dependent on CD25 or miR-15/16 expression. Venn diagrams were generated using Venny 2.1 (https://bioinfogp.cnb.csic.es/tools/venny/). Gene Set Enrichment Analysis (www.gsea-msigdb.org) was performed on 300 most upregulated or 300 most downregulated genes in Tregs with constitutive STAT5b expression compared to WT Tregs, and plotted against gene expression across miR-15/16 deficient or sufficient CD4^+^ T cells.^22^ Gene Set Enrichment Analysis was also performed on upregulated genes in miR-15/16 deficient Tregs compared to WT Tregs, and AHC >5 at miR-15/16 seed-matches in CD4^+^ T cells,^22^ plotted against the gene expression profile of eTreg or rTreg subsets.^11^ The cumulative distribution function plot was generated in R (www.r-project.org) to display the log2 fold change of DEGs (miR-15/16 deficient versus miR-15/16 sufficient Tregs) against the cumulative distribution of genes. The expression values from all genes lacking miR-15/16 3′UTR seed-match were plotted along with all genes containing a miR-15/16 3′UTR seed-match, a subset of all genes containing a miR-15/16 3′UTR seed-match with >5 AHC read mapping to the seed-match, and all genes predicted to be targeted by miR-15/16 by TargetScan (www.targetscan.org).

### QUANTIFICATION AND STATISTICAL ANALYSIS

Microsoft Excel and GraphPad Prism 9 were used for data analysis. In all figures, bar graphs are displayed with error bars showing standard deviation unless otherwise stated. *Z* score was calculated from mean and standard deviation. *p < 0.05, **p < 0.01, ***p < 0.001, and ****p < 0.0001 for significance. ANOVA was used as statistical test of multiple groups, with appropriate post hoc testing as stated in the text. All data was assumed to be normally distributed.

### ADDITIONAL RESOURCES

This study has previously been reported in the form of a preprint (https://doi.org/10.1101/2023.03.26.533356).

## Supplementary Material

1

2

## Figures and Tables

**KEY RESOURCES TABLE:** 

REAGENT or RESOURCE	SOURCE	IDENTIFIER

**Antibodies**		

Anti-CD3 PE-CF594 (145-2C11)	BioLegend	Cat# 100348; RRID: AB_2564029
Anti-CD4 FITC (GK1.5)	Thermo Fisher Scientific	Cat# 11-0041-82; RRID: AB_464892
Anti-CD4 APC (GK1.5)	Thermo Fisher Scientific	Cat# 17-0041-83; RRID: AB_469321
Anti-CD4 AF700 (GK1.5)	Thermo Fisher Scientific	Cat# 56-0041-82; RRID: AB_493999
Anti-CD8a PE (53-6.7)	BioLegend	Cat# 100707; RRID: AB_312747
Anti-CD8a BV605 (53-6.7)	BioLegend	Cat# 100744; RRID: AB_2562609
Anti-CD11b PerCP-Cy5.5 (M1/70)	BioLegend	Cat# 101228; RRID: AB_893233
Anti-CD11c AF700 (N418)	BioLegend	Cat# 117320; RRID: AB_528736
Anti-CD19 PE-Cy7 (1D3)	Thermo Fisher Scientific	Cat# 25-0193-82; RRID: AB_528736
Anti-CD25 PE-Cy7 (PC61.5)	Thermo Fisher Scientific	Cat# 25-0251-82; RRID: AB_469608
Anti-CD25 APC (PC61.5)	Thermo Fisher Scientific	Cat# 17-0251-82; RRID: AB_469366
Anti-CD44 PE-CF594 (IM7)	BioLegend	Cat# 103055; RRID: AB_2564043
Anti-CD44 PE-Cy7 (IM7)	BioLegend	Cat# 103030; RRID: AB_830786
Anti-CD45 BV510 (30-F11)	BD Biosciences	Cat# 563891; RRID: AB_2734134
Anti-CD45 BV786 (30-F11)	BD Biosciences	Cat# 564225; RRID: AB_2716861
Anti-CD45.1 BV510(A20)	BioLegend	Cat# 110741; RRID: AB_2563378
Anti-CD45.1 BV605 (A20)	BioLegend	Cat# 110737; RRID: AB_11204076
Anti-CD45.1 PE (A20)	BioLegend	Cat# 110707; RRID: AB_313497
Anti-CD45.2 PE-Cy7 (104)	Thermo Fisher Scientific	Cat# 25-0454-82; RRID: AB_2573350
Anti-CD45.2 AF700 (104)	Thermo Fisher Scientific	Cat# 56-0454-81; RRID: AB_657753
Anti-CD62L (MEL-14) BV605	BioLegend	Cat# 104438; RRID: AB_11125577
Anti-CD62L (MEL-14) FITC	BioLegend	Cat# 104405; RRID: AB_313092
Anti-CD62L (MEL-14) PE-Cy7	BioLegend	Cat# 104417; RRID: AB_313103
Anti-CD122 PE (5H4)	BioLegend	Cat# 105905; RRID: AB_2125736
Anti-CD127 FITC (A7R34)	Thermo Fisher Scientific	Cat# 11-1271-82; RRID: AB_465195
Anti-CD127 BV421 (A7R34)	BioLegend	Cat# 135024; RRID: AB_11218800
Anti-CD132 APC (TUGm2)	BioLegend	Cat# 132307; RRID: AB_10643576
Anti-CD152 PerCP-Cy5.5 (UC10-4B9)	BioLegend	Cat# 106316; RRID: AB_2564473
Anti-CD279 BV785 (29F.1A12)	BioLegend	Cat# 135225; RRID: AB_2563680
Anti-CD279 PE-CF594 (29F.1A12)	BioLegend	Cat# 135228; RRID: AB_2566005
Anti-CD279 APC (29F.1A12)	BioLegend	Cat# 135209; RRID: AB_2159183
Anti-CCR6 PE (29-2L17)	BioLegend	Cat# 129804; RRID: AB_1279139
Anti-Ly6G eFluor 450 (1A8-Ly6g)	Thermo Fisher Scientific	Cat# 48-9668-82; RRID: AB_2637124
Anti-NK1.1 AF647 (PK136)	BioLegend	Cat# 108719; RRID: AB_2132713
Anti-NRP-1 APC (3E12)	BioLegend	Cat# 145206; RRID: AB_2562031
Anti-Siglec-F PE (E50-2440)	BD Biosciences	Cat# 552126; RRID: AB_10896143
Anti-ST2 BV605 (U29-93)	BD Biosciences	Cat# 745257; RRID:AB_2742841
Anti-FOXP3 BV421 (FJK-16s)	Thermo Fisher Scientific	Cat# 404-5773-82; RRID: AB_2925536
Anti-FOXP3 FITC (FJK-16s)	Thermo Fisher Scientific	Cat# 11-5773-82; RRID: AB_465243
Anti-FOXP3 APC (FJK-16s)	Thermo Fisher Scientific	Cat# 17-5773-82; RRID: AB_469457
Anti-TBET PE (4B10)	BioLegend	Cat# 644809; RRID: AB_2028583
Anti-RORγT BV786 (Q31-378)	BD Biosciences	Cat# 564723; RRID: AB_2738916
Anti-HELIOS PE (22F6)	BioLegend	Cat# 137216; RRID: AB_10552903
Anti-IL-2 PE (JES6-5H4)	BioLegend	Cat# 503807; RRID: AB_315302
Anti-IL-10 APC (JES5-16E3)	Thermo Fisher Scientific	Cat# 17-7101-82; RRID: AB_469502
Anti-IL-13 PE-Cy7 (eBio13A)	Thermo Fisher Scientific	Cat# 25-7133-82; RRID: AB_2573530
Anti-IL-17A eFluor 450 (eBio17B7)	Thermo Fisher Scientific	Cat# 48-7177-82; RRID: AB_11149503
Anti-IFNg PerCP-Cy5.5 (XMG1.2)	Thermo Fisher Scientific	Cat# 45-7311-82; RRID: AB_1107020
Anti-STAT5 pY694 (47) PE-Cy7	BD Biosciences	Cat# 560117; RRID: AB_1645546
Anti-CD3 (coating) (2C11)	Bio X Cell	Cat# BP0001-1; RRID: AB_1107634
Anti-CD28 (coating) (37.51)	Bio X Cell	Cat# BE0015-1; RRID: AB_1107624
Anti-CD16/CD32 (2.4G2)	Bio X Cell	Cat# BE0307; RRID: AB_2736987

**Chemicals, peptides, and recombinant proteins**		

Ovalbumin	Sigma-Aldrich	Cat# A5503
Imject Alum adjuvant	Thermo Fisher Scientific	Cat# 77161
Recombinant IL-2	National Cancer Institute	NA
Recombinant IL-7	R&D systems	Cat# 407-ML-200/CF
Saponin	Sigma-Aldrich	Cat# 47036-50G-F
Mitomycin C	Roche	Cat# ROCH10107409001

**Critical commercial assays**		

Fixable Viability Dye eFluor 780	Thermo Fisher Scientific	Cat# 65-0865-14
Click-iT Plus EdU AF647 Flow cytometry assay kit (including EdU)	Thermo Fisher Scientific	Cat# C10634
Foxp3/Transcription Factor Staining Buffer set	Thermo Fisher Scientific	Cat# 00-5523-00
EasySep^™^ Mouse Naive CD4^+^ T cell isolation kit	STEMCELL Technologies	Cat# 19765
EasySep^™^ Mouse CD4^+^ T cell isolation kit	STEMCELL Technologies	Cat# 19852
AccuCount blank particles	Spherotech Inc	Cat# ACBP-100-10
UltraComp eBeads^™^ compensation beads	Thermo Fisher Scientific	Cat# 01-2222-42
Invitrogen^™^ CellTrace^™^ Violet Cell Proliferation kit	Fisher Scientific	Cat# 15579992

**Deposited data**		

RNA-sequencing	This study	GEO: GSE230099

**Experimental models: Organisms/strains**		

B6.Cg-Tg(CD4-cre)1Cwi	Taconic	Cd4-cre Model# 4196
B6.129P2-*Tcrb^tm1Mom^/J*	The Jackson Laboratory	Tcrb KO Stain# 002118
B6.SJL-PtprcaPepcb/BoyCrCrl	Charles River	CD45.1 Strain# 564
B6.Cg-*Foxp3^tm2Tch^*/J	The Jackson Laboratory	FOXP3-GFP Strain# 006772
B6.129(Cg)-*Foxp3^tm4(YFP/icre)Ayr^*/J	The Jackson Laboratory	*Foxp3*^Cre^ YFP Strain# 016959
B6.129S-*Mirc30^tm1.1Rdf^*/J	The Jackson Laboratory	miR-15a/16-1 fl Strain# 017641
*Mirc10^tm1Mtm^*/Mmjax	The Jackson Laboratory	miR-15b/16-2 fl MMRRC Stock# 37643-JAX

**Software and algorithms**		

FlowJo v10	FlowJo, LCC	NA
Prism 9	GraphPad Software	www.graphpad.com
Ingenuity Pathway Analysis	Qiagen	NA
R	The R Foundation	www.r-project.org
Venny 2.1	BioinfoGP	https://bioinfogp.cnb.csic.es/tools/venny/
ImageJ	Schneider et al.^54^	https://ImageJ.nih.gov/ij/

## Highlights

- Treg-specific miR-15/16 expression is essential to prevent systemic tissue inflammation
- miR-15/16 restrict Treg proliferation and regulate expression of key functional molecules
- miR-15/16 limit formation of effector Tregs and are necessary for high suppressive capacity

## INCLUSION AND DIVERSITY

We support inclusive, diverse, and equitable conduct of research.

## References

[1] Fontenot JD, Rasmussen JP, Gavin MA, and Rudensky AY (2005). A function for interleukin 2 in Foxp3-expressing regulatory T cells. Nat. Immunol. 6, 1142–1151. 10.1038/ni1263. 16227984

[2] Liston A, Farr AG, Chen Z, Benoist C, Mathis D, Manley NR, and Rudensky AY (2007). Lack of Foxp3 function and expression in the thymic epithelium. J. Exp. Med. 204, 475–480. 10.1084/jem.20062465. 17353370 PMC2137899

[3] Rudensky AY (2011). Regulatory T Cells and Foxp3. Immunol. Rev. 241, 260–268. 10.1111/j.1600-065X.2011.01018.x. 21488902 PMC3077798

[4] Feng Y, Arvey A, Chinen T, van der Veeken J, Gasteiger G, and Rudensky AY (2014). Control of the inheritance of regulatory T cell identity by a cis element in the Foxp3 locus. Cell 158, 749–763. 10.1016/j.cell.2014.07.031. 25126783 PMC4151558

[5] Li X, Liang Y, LeBlanc M, Benner C, and Zheng Y (2014). Function of a Foxp3 cis-element in protecting regulatory T cell identity. Cell 158, 734–748. 10.1016/j.cell.2014.07.030. 25126782 PMC4151505

[6] Chinen T, Kannan AK, Levine AG, Fan X, Klein U, Zheng Y, Gasteiger G, Feng Y, Fontenot JD, and Rudensky AY (2016). An essential role for IL-2 receptor in regulatory T cell function. Nat. Immunol. 17, 1322–1333. 10.1038/ni.3540. 27595233 PMC5071159

[7] Toomer KH, Lui JB, Altman NH, Ban Y, Chen X, and Malek TR (2019). Essential and non-overlapping IL-2Rα-dependent processes for thymic development and peripheral homeostasis of regulatory T cells. Nat. Commun. 10, 1037. 10.1038/s41467-019-08960-1. 30833563 PMC6399264

[8] Lio CWJ, and Hsieh CS (2008). A Two-Step Process for Thymic Regulatory T Cell Development. Immunity 28, 100–111. 10.1016/j.immuni.2007.11.021. 18199417 PMC2248212

[9] Tai X, Erman B, Alag A, Mu J, Kimura M, Katz G, Guinter T, McCaughtry T, Etzensperger R, Feigenbaum L, (2013). Foxp3 Transcription Factor Is Proapoptotic and Lethal to Developing Regulatory T Cells unless Counterbalanced by Cytokine Survival Signals. Immunity 38, 1116–1128. 10.1016/j.immuni.2013.02.022. 23746651 PMC3700677

[10] Burchill MA, Yang J, Vang KB, Moon JJ, Chu HH, Lio CWJ, Vegoe AL, Hsieh CS, Jenkins MK, and Farrar MA (2008). Linked T Cell Receptor and Cytokine Signaling Govern the Development of the Regulatory T Cell Repertoire. Immunity 28, 112–121. 10.1016/j.immuni.2007.11.022. 18199418 PMC2430111

[11] Yang BH, Wang K, Wan S, Liang Y, Yuan X, Dong Y, (2019). TCF1 and LEF1 Control Treg Competitive Survival and Tfr Development to Prevent Autoimmune Diseases. Cell Rep. 27, 3629–3645.e6. 10.1016/j.celrep.2019.05.061. 31216480 PMC6701704

[12] Sjaastad LE, Owen DL, Tracy SI, and Farrar MA (2021). Phenotypic and Functional Diversity in Regulatory T Cells. Front. Cell Dev. Biol. 9, 715901. 10.3389/fcell.2021.715901. 34631704 PMC8495164

[13] Xing S, Gai K, Li X, Shao P, Zeng Z, Zhao X, Zhao X, Chen X, Paradee WJ, Meyerholz DK, (2019). Tcf1 and Lef1 are required for the immunosuppressive function of regulatory T cells. J. Exp. Med. 216, 847–866. 10.1084/jem.20182010. 30837262 PMC6446865

[14] Bartel DP (2004). MicroRNAs: Genomics, Biogenesis, Mechanism, and Function. Cell 116, 281–297. 10.1016/s0092-8674(04)00045-5. 14744438

[15] Cobb BS, Hertweck A, Smith J, O’Connor E, Graf D, Cook T, Smale ST, Sakaguchi S, Livesey FJ, Fisher AG, and Merkenschlager M (2006). A role for Dicer in immune regulation. J. Exp. Med. 203, 2519–2527. 10.1084/jem.20061692. 17060477 PMC2118134

[16] Liston A, Lu LF, O’Carroll D, Tarakhovsky A, and Rudensky AY (2008). Dicer-dependent microRNA pathway safeguards regulatory T cell function. J. Exp. Med. 205, 1993–2004. 10.1084/jem.20081062. 18725526 PMC2526195

[17] Zhou X, Jeker LT, Fife BT, Zhu S, Anderson MS, McManus MT, and Bluestone JA (2008). Selective miRNA disruption in T reg cells leads to uncontrolled autoimmunity. J. Exp. Med. 205, 1983–1991. 10.1084/jem.20080707. 18725525 PMC2526194

[18] Jeker LT, and Bluestone JA (2013). microRNA regulation of T-cell differentiation and function. Immunol. Rev. 253, 65–81. 10.1111/imr.12061. 23550639 PMC3621017

[19] Podshivalova K, and Salomon DR (2013). microRNA regulation of T lymphocyte immunity: modulation of molecular networks responsible for T cell activation, differentiation and development. Crit. Rev. Immunol. 33, 435–476. 10.1615/critrevimmunol.2013006858. 24099302 PMC4185288

[20] Calin GA, Sevignani C, Dumitru CD, Hyslop T, Noch E, Yendamuri S, (2004). Human microRNA genes are frequently located at fragile sites and genomic regions involved in cancers. Proc Natl Acad Sci USA 101, 2999–3004. 10.1073/pnas.0307323101. 14973191 PMC365734

[21] Liu T, Xu Z, Ou D, Liu J, and Zhang J (2019). The miR-15a/16 gene cluster in human cancer: A systematic review. J. Cell. Physiol. 234, 5496–5506. 10.1002/jcp.27342. 30246332

[22] Gagnon JD, Kageyama R, Shehata HM, Fassett MS, Mar DJ, Wigton EJ, Johansson K, Litterman AJ, Odorizzi P, Simeonov D, (2019). miR-15/16 Restrain Memory T Cell Differentiation, Cell Cycle, and Survival. Cell Rep. 28, 2169–2181.e4. 10.1016/j.celrep.2019.07.064. 31433990 PMC6715152

[23] Urena F, Ma C, Hoffmann FW, Nunes LGA, Urschitz J, Moisyadi S, Khadka VS, Deng Y, and Hoffmann PR (2022). T-cell activation decreases miRNA-15a/16 levels to promote MEK1–ERK1/2–Elk1 signaling and proliferative capacity. J. Biol. Chem. 298, 101634. 10.1016/j.jbc.2022.101634. 35085550 PMC8861121

[24] Marcais A, Blevins R, Graumann J, Feytout A, Dharmalingam G, Carroll T, Amado IF, Bruno L, Lee K, Walzer T, (2014). micro-RNA-mediated regulation of mTOR complex components facilitates discrimination between activation and anergy in CD4 T cells. J. Exp. Med. 211, 2281–2295. 10.1084/jem.20132059. 25311506 PMC4203951

[25] Wu YH, Liu W, Xue B, Zhang L, Liu XY, Liu B, Wang Y, Cai Y, and Duan R (2016). Upregulated Expression of microRNA-16 Correlates with Th17/Treg Cell Imbalance in Patients with Rheumatoid Arthritis. DNA Cell Biol. 35, 853–860. 10.1089/dna.2016.3349. 27875659

[26] Yang J, Liu R, Deng Y, Qian J, Lu Z, Wang Y, Zhang D, Luo F, and Chu Y (2017). MiR-15a/16 deficiency enhances anti-tumor immunity of glioma-infiltrating CD8+ T cells through targeting mTOR. Int. J. Cancer 141, 2082–2092. 10.1002/ijc.30912. 28758198

[27] Singh Y, Garden OA, Lang F, and Cobb BS (2015). MicroRNA-15b/16 Enhances the Induction of Regulatory T Cells by Regulating the Expression of Rictor and mTOR. J. Immunol. 195, 5667–5677. 10.4049/jimmunol.1401875. 26538392 PMC4671309

[28] Dong J, Huth WJ, Marcel N, Zhang Z, Lin LL, and Lu LF (2023). miR-15/16 clusters restrict effector Treg cell differentiation and function. J. Exp. Med. 220, e20230321. 10.1084/jem.20230321. 37516921 PMC10374942

[29] Rossi RL, Rossetti G, Wenandy L, Curti S, Ripamonti A, Bonnal RJP, Birolo RS, Moro M, Crosti MC, Gruarin P, (2011). Distinct microRNA signatures in human lymphocyte subsets and enforcement of the naive state in CD4+ T cells by the microRNA miR-125b. Nat. Immunol. 12, 796–803. 10.1038/ni.2057. 21706005

[30] Yadav M, Louvet C, Davini D, Gardner JM, Martinez-Llordella M, Bailey-Bucktrout S, Anthony BA, Sverdrup FM, Head R, Kuster DJ, (2012). Neuropilin-1 distinguishes natural and inducible regulatory T cells among regulatory T cell subsets in vivo. J. Exp. Med. 209, 1713–1722, S1–19. 10.1084/jem.20120822. 22966003 PMC3457729

[31] Nelson BH (2004). IL-2, Regulatory T Cells, and Tolerance. J. Immunol. 172, 3983–3988. 10.4049/jimmunol.172.7.3983. 15034008

[32] Katzman SD, Hoyer KK, Dooms H, Gratz IK, Rosenblum MD, Paw JS, Isakson SH, and Abbas AK (2011). Opposing functions of IL-2 and IL-7 in the regulation of immune responses. Cytokine 56, 116–121. 10.1016/j.cyto.2011.07.005. 21807532 PMC3171642

[33] Sakaguchi S, Mikami N, Wing JB, Tanaka A, Ichiyama K, and Ohkura N (2020). Regulatory T Cells and Human Disease. Annu. Rev. Immunol. 38, 541–566. 10.1146/annurev-immunol-042718-041717. 32017635

[34] Liston A, and Gray DHD (2014). Homeostatic control of regulatory T cell diversity. Nat. Rev. Immunol. 14, 154–165. 10.1038/nri3605. 24481337

[35] Panduro M, Benoist C, and Mathis D (2016). TISSUE-Tregs. Annu. Rev. Immunol. 34, 609–633. 10.1146/annurev-immunol-032712-095948. 27168246 PMC4942112

[36] Campbell DJ (2015). Control of Regulatory T Cell Migration, Function, and Homeostasis. J. Immunol. 195, 2507–2513. 10.4049/jimmunol.1500801. 26342103 PMC4778549

[37] Cretney E, Kallies A, and Nutt SL (2013). Differentiation and function of Foxp3(+) effector regulatory T cells. Trends Immunol. 34, 74–80. 10.1016/j.it.2012.11.002. 23219401

[38] Pua HH, Steiner DF, Patel S, Gonzalez JR, Ortiz-Carpena JF, Kageyama R, Chiou NT, Gallman A, de Kouchkovsky D, Jeker LT, (2016). MicroRNAs 24 and 27 suppress allergic inflammation and target a network of regulators of T helper-2 cell-associated cytokine production. Immunity 44, 821–832. 10.1016/j.immuni.2016.01.003. 26850657 PMC4838571

[39] Simpson LJ, Patel S, Bhakta NR, Choy DF, Brightbill HD, Ren X, Wang Y, Pua HH, Baumjohann D, Montoya MM, (2014). A miRNA upregulated in asthma airway T cells promotes TH2 cytokine production. Nat. Immunol. 15, 1162–1170. 10.1038/ni.3026. 25362490 PMC4233009

[40] Bai J, Gao Z, Li X, Dong L, Han W, and Nie J (2017). Regulation of PD-1/PD-L1 pathway and resistance to PD-1/PD-L1 blockade. Oncotarget 8, 110693–110707. 10.18632/oncotarget.22690. 29299180 PMC5746415

[41] Lowther DE, Goods BA, Lucca LE, Lerner BA, Raddassi K, van Dijk D, (2016). PD-1 marks dysfunctional regulatory T cells in malignant gliomas. JCI Insight 1, e85935. 10.1172/jci.insight.85935. 27182555 PMC4864991

[42] Klein U, Lia M, Crespo M, Siegel R, Shen Q, Mo T, Ambesi-Impiombato A, Califano A, Migliazza A, Bhagat G, and Dalla-Favera R (2010). The DLEU2/miR-15a/16–1 Cluster Controls B Cell Proliferation and Its Deletion Leads to Chronic Lymphocytic Leukemia. Cancer Cell 17, 28–40. 10.1016/j.ccr.2009.11.019. 20060366

[43] Mazzucchelli R, Hixon JA, Spolski R, Chen X, Li WQ, Hall VL, Willette-Brown J, Hurwitz AA, Leonard WJ, and Durum SK (2008). Development of regulatory T cells requires IL-7Rα stimulation by IL-7 or TSLP. Blood 112, 3283–3292. 10.1182/blood-2008-02-137414. 18664628 PMC2569178

[44] Fan MY, Low JS, Tanimine N, Finn KK, Priyadharshini B, Germana SK, Kaech SM, and Turka LA (2018). Differential Roles of IL-2 Signaling in Developing versus Mature Tregs. Cell Rep. 25, 1204–1213.e4. 10.1016/j.celrep.2018.10.002. 30380412 PMC6289175

[45] Bayer AL, Lee JY, de la Barrera A, Surh CD, and Malek TR (2008). A Function for IL-7R for CD4+CD25+Foxp3+ T Regulatory Cells. J. Immunol. 181, 225–234. 10.4049/jimmunol.181.1.225. 18566388 PMC2601574

[46] Vang KB, Yang J, Mahmud SA, Burchill MA, Vegoe AL, and Farrar MA (2008). IL-2, −7, and −15, but Not Thymic Stromal Lymphopoeitin, Redundantly Govern CD4+Foxp3+ Regulatory T Cell Development. J. Immunol. 181, 3285–3290. 10.4049/jimmunol.181.5.3285. 18714000 PMC2810104

[47] Li G, Miskimen KL, Wang Z, Xie XY, Brenzovich J, Ryan JJ, Tse W, Moriggl R, and Bunting KD (2010). STAT5 requires the N-domain for suppression of miR15/16, induction of bcl-2, and survival signaling in myeloproliferative disease. Blood 115, 1416–1424. 10.1182/blood-2009-07-234963. 20008792 PMC2826763

[48] Nishioka T, Shimizu J, Iida R, Yamazaki S, and Sakaguchi S (2006). CD4+CD25+Foxp3+ T cells and CD4+CD25-Foxp3+ T cells in aged mice. J. Immunol. 176, 6586–6593. 10.4049/jimmunol.176.11.6586. 16709816

[49] Raynor J, Sholl A, Plas DR, Bouillet P, Chougnet CA, and Hildeman DA (2013). IL-15 Fosters Age-Driven Regulatory T Cell Accrual in the Face of Declining IL-2 Levels. Front. Immunol. 4, 161. 10.3389/fimmu.2013.00161. 23805138 PMC3690359

[50] Freimer JW, Shaked O, Naqvi S, Sinnott-Armstrong N, Kathiria A, Garrido CM, Chen AF, Cortez JT, Greenleaf WJ, Pritchard JK, and Marson A (2022). Systematic discovery and perturbation of regulatory genes in human T cells reveals the architecture of immune networks. Nat. Genet. 54, 1133–1144. 10.1038/s41588-022-01106-y. 35817986 PMC10035359

[51] Danilo M, Chennupati V, Silva JG, Siegert S, and Held W (2018). Suppression of Tcf1 by Inflammatory Cytokines Facilitates Effector CD8 T Cell Differentiation. Cell Rep. 22, 2107–2117. 10.1016/j.celrep.2018.01.072. 29466737

[52] Yu Q, Erman B, Park JH, Feigenbaum L, and Singer A (2004). IL-7 Receptor Signals Inhibit Expression of Transcription Factors TCF-1, LEF-1, and RORγt : Impact on Thymocyte Development. J. Exp. Med. 200, 797–803. 10.1084/jem.20032183. 15365098 PMC2211960

[53] Barra MM, Richards DM, Hansson J, Hofer AC, Delacher M, Hettinger J, Krijgsveld J, and Feuerer M (2015). Transcription Factor 7 Limits Regulatory T Cell Generation in the Thymus. J. Immunol. 195, 3058–3070. 10.4049/jimmunol.1500821. 26324778

[54] Schneider CA, Rasband WS, and Eliceiri KW (2012). NIH Image to ImageJ: 25 years of image analysis. Nat. Methods 9, 671–675. 10.1038/nmeth.2089. 22930834 PMC5554542

[55] Villarino A, Laurence A, Robinson GW, Bonelli M, Dema B, Afzali B, Shih HY, Sun HW, Brooks SR, Hennighausen L, (2016). Signal transducer and activator of transcription 5 (STAT5) paralog dose governs T cell effector and regulatory functions. Elife 5, e08384. 10.7554/eLife.08384. 26999798 PMC4856466

