# Supplementary material for: An essential role for miR-15/16 in Treg suppression and restriction of proliferation
Source: Cell Rep. Author manuscript; Available in PMC 2023 Nov 22. (PMC10664750; doi:10.1016/j.celrep.2023.113298)
Supplement: 1 [file NIHMS1941905-supplement-1.pdf]

Cell Reports, Volume *42*

Supplemental information

An essential role for miR-15/16 in Treg suppression and restriction of proliferation

Kristina Johansson, John D. Gagnon, Simon K. Zhou, Marlys S. Fassett, Andrew W. Schroeder, Robin Kageyama, Rodriel A. Bautista, Hewlett Pham, Prescott G. Woodruff, and K. Mark Ansel

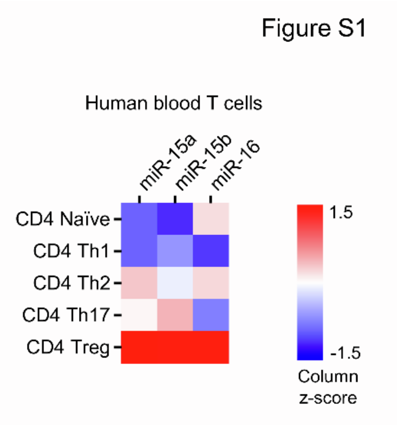

Supplementary Figure S1. miR-15/16 expression in human T cell subsets, Related to Figure 1.
qPCR of miRNAs purified from $CD4^+$ T cell subsets in peripheral blood of healthy donors (Rossi *et al.* [S1]; GSE22880). Flow cytometry was used for isolation of T cell subsets; CD4 Naïve $CD4^+CCR7^+CD45RA^+CD45RO^-$; CD4 Th1 $CD4^+CXCR3^+CCR6^-CD161^-$; CD4 Th2 $CD4^+CRTH2^+CXCR3^-$; CD4 Th17 $CD4^+CCR6^+CD161^+CXCR3^-$; CD4 Treg $CD4^+CD127^{lo}CD25^+$. N=3-6/group.

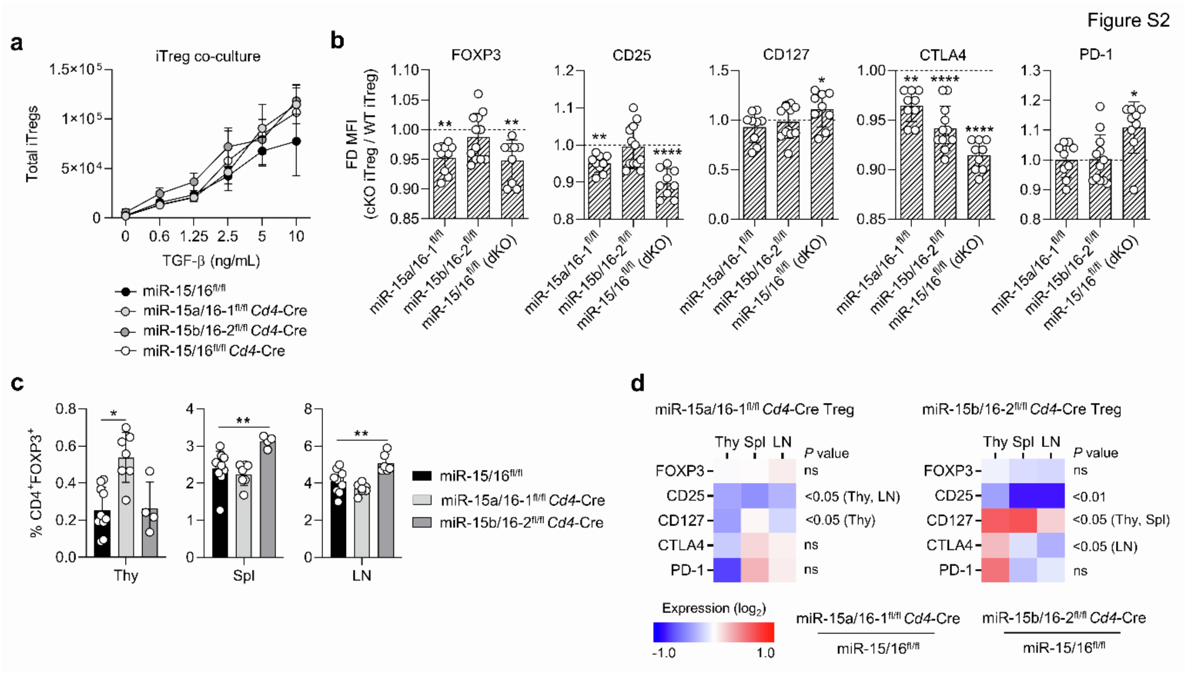

Supplementary Figure S2. miR-15/16 single cluster expression modulate Treg phenotype, Related to Figure 1.

A: Total number of induced Tregs (iTregs) after 5 days in co-culture under Treg polarizing conditions assessed by FOXP3 expression by flow cytometry. **B:** Protein expression by median fluorescent intensity (MFI) compared to co-cultured WT control iTregs. **C:** Frequency of Tregs among all T cells of miR-15a/16-1$^{fl/fl}$ *Cd4*-Cre mice, miR-15b/16-2$^{fl/fl}$ *Cd4*-Cre mice and miR-15a/16-1$^{fl/fl}$ control mice. **D:** Fold change of MFI by flow cytometry of indicated proteins in Tregs of miR-15a/16-1$^{fl/fl}$ *Cd4*-Cre mice (left) and miR-15b/16-2$^{fl/fl}$ *Cd4*-Cre mice (right) from three tissues (change in knockout from miR-15/16$^{fl/fl}$ WT control). Data from a minimum of 2 independent experiments. N=5-12 mice/group. Ordinary ANOVA with Dunnett's multiple comparison test in B-C. Bar graphs are shown with error bars demonstrating standard deviation.

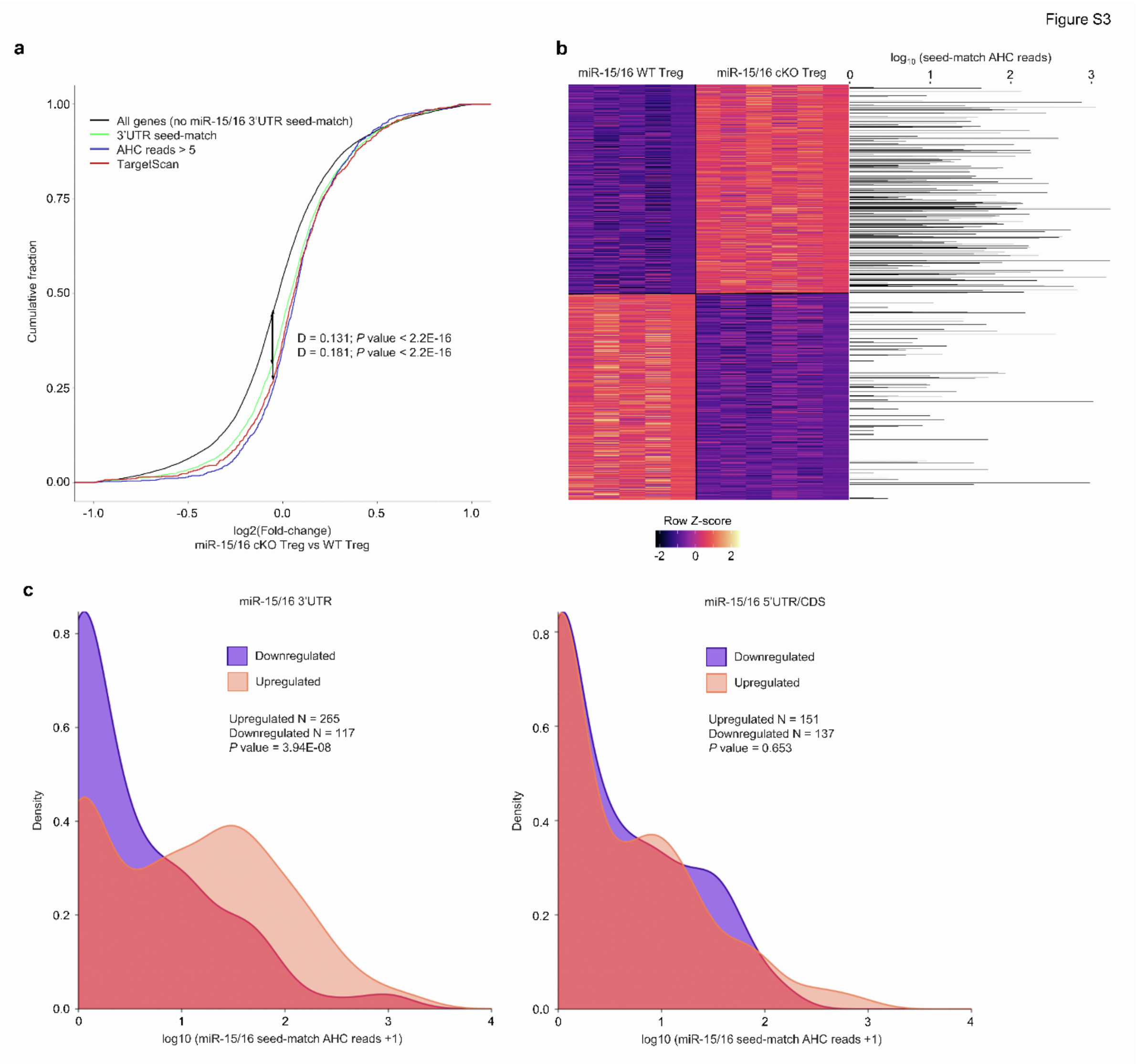

Supplementary Figure S3. miR-15/16 bind and regulate direct target RNAs in Tregs, Related to Figure 7.

A: Cumulative density plot depicting global expression by RNA sequencing as a ratio of the fold change between miR-15/16$^{fl/fl}$ *Foxp3*^{Cre} ('miR-15/16 cKO Treg'; n = 6 biological replicates) and miR-15/16$^{wt/wt}$ *Foxp3*^{Cre} ('miR-15/16 WT Treg'; n = 5 biological replicates) Tregs for all genes without a 7-mer or 8-mer miR-15/16 3'UTR seed match (black), genes with a 7-mer or 8- mer miR-15/16 3'UTR seed match (green), genes with a 7-mer or 8-mer miR-15/16 3'UTR seed match and AHC read depth >5 (blue), and genes classified as targets of miR-15/16 by TargetScan 7.0 (red) (AHC reads represent the combined depth of n = 10 independent immunoprecipitations). **B:** Heatmap of genes with a P-value <0.05 plotted alongside a bar graph of AHC read depth at miR-15/16 seed matches for each gene at which they occur. **C:** Comparison of AHC reads between genes that are downregulated and upregulated (P<0.05) in miR-15/16$^{fl/fl}$ *Foxp3*^{Cre} Tregs among genes with seed matches in the 3'UTR (left) or 5'UTR/CDS (right) (Mann-Whitney U test). AHC data was generated by Gagnon *et al.* [S2]; GSE111568.

Figure S4

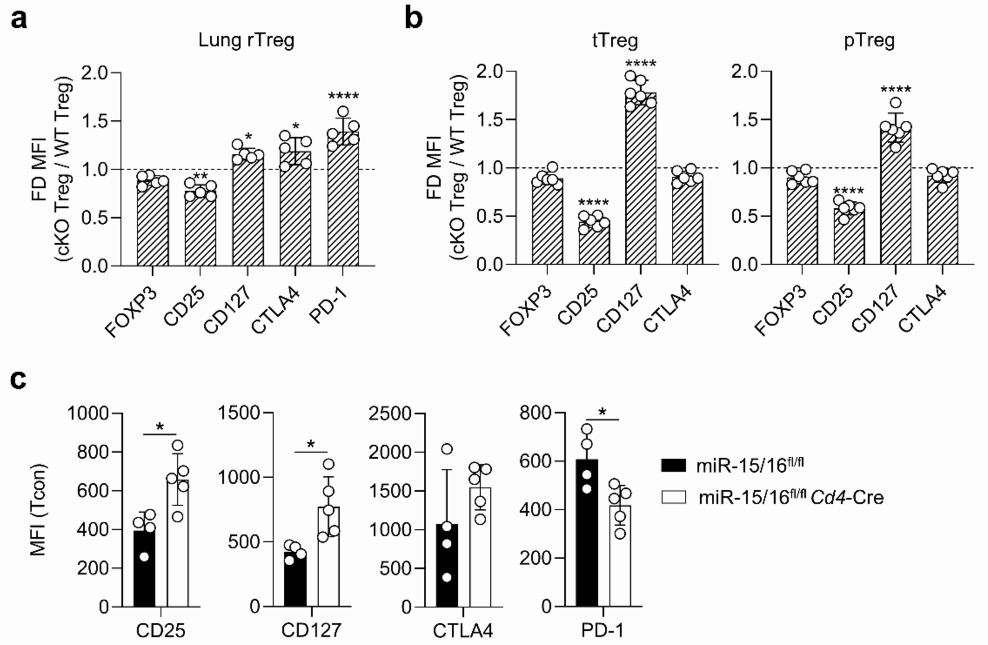

Supplementary Figure S4. Treg subset phenotype, Related to Figure 1.
Fold difference of expression by median fluorescent intensity (MFI) in miR-15/16$^{fl/fl}$ and miR-15/16$^{fl/fl}$ *Cd4*-Cre Tregs (change in miR-15/16$^{fl/fl}$ *Cd4*-Cre from miR-15/16$^{fl/fl}$ WT control) in lung tissue resident Tregs **(A)** and thymic (tTreg, Helios^{+}NRP-1^{+}) and peripheral (pTreg, Helios^{-}NRP-1^{-}) Tregs **(B)** in spleen**. C:** Receptor MFI values in CD4^{+} conventional T cells (non-Treg) infiltrating the airways (bronchoalveolar lavage) after OVA-induced inflammation (model in Figure 1L) in miR-15/16$^{fl/fl}$ *Cd4*-Cre from miR-15/16$^{fl/fl}$ WT control mice. Data from 2 independent experiments. N=4-5/group. Ordinary ANOVA with Dunnett's multiple comparison test in A-B. Unpaired t-test 2-tailed in C. Bar graphs are shown with error bars demonstrating standard deviation.

References

[S1]. Rossi RL, Rossetti G, Wenandy L, Curti S, Ripamonti A, Bonnal RJP, et al. (2011). Distinct microRNA signatures in human lymphocyte subsets and enforcement of the naive state in CD4+ T cells by the microRNA miR-125b. Nat Immunol 12, 796–803. doi: 10.1038/ni.2057.

[S2]. Gagnon JD, Kageyama R, Shehata HM, Fassett MS, Mar DJ, Wigton EJ, et al. (2019). miR-15/16 Restrain Memory T Cell Differentiation, Cell Cycle, and Survival. Cell Rep 28, 2169-2181.e4. doi: 10.1016/j.celrep.2019.07.064.
